# A Viral RNA Silencing Suppressor Modulates Reactive Oxygen Species Levels to Induce the Autophagic Degradation of Dicer‐Like and Argonaute‐Like Proteins

**DOI:** 10.1002/advs.202506572

**Published:** 2025-09-08

**Authors:** Shiyu Zhai, Tianxing Pang, Shiyu Peng, Shenshen Zou, Zhiping Deng, Nobuhiro Suzuki, Zhensheng Kang, Ida Bagus Andika, Liying Sun

**Affiliations:** ^1^ State Key Laboratory of Crop Stress Biology for Arid Areas and College of Plant Protection Northwest A&F University Yangling Shaanxi 712100 P. R. China; ^2^ Department of Plant Pathology, College of Plant Protection Shandong Agricultural University Tai'an 271002 P. R. China; ^3^ Institute of Virology and Biotechnology Zhejiang Academy of Agricultural Sciences Hangzhou Zhejiang 310021 P. R. China; ^4^ Institute of Plant Science and Resources (IPSR) Okayama University Kurashiki 710‐0046 Japan; ^5^ Institute of Future Agriculture Northwest A&F University Yangling Shaanxi 712100 P. R. China

**Keywords:** argonaute, autophagic degradation, cryphonectria hypovirus 1, dicer, reactive oxygen species, RNA silencing suppressor

## Abstract

Mounting evidence indicates that viruses exploit elevated reactive oxygen species (ROS) levels to promote replication and pathogenesis, yet the mechanistic underpinnings of this viral strategy remain elusive for many viral systems. This study uncovers a sophisticated viral counter‐defense mechanism in the Cryphonectria hypovirus 1 (CHV1)‐*Fusarium graminearum* system, where the viral p29 protein subverts host redox homeostasis to overcome antiviral responses. That p29 directly interacts with and inhibits the enzymatic activity of fungal NAD(P)H‐dependent FMN reductase 1 (FMR1), leading to increased ROS accumulation and subsequent autophagy activation is demonstrated. Strikingly, this ROS‐induced autophagy selectively targets for degradation two core antiviral RNA silencing components against CHV1 in *F. graminearum*, Dicer‐like 2 (DCL2) and Argonaute‐like 1 (AGL1), thereby compromising the host's primary antiviral defense system. Genetic analysis confirms this coordinated hijacking of host machineries, as CHV1 shows enhanced accumulation in the *FMR1* knockout and reduced accumulation in autophagy‐deficient fungal strains. This work reveals a tripartite interplay among oxidative stress, autophagy, and RNA silencing that CHV1 manipulates through p29 multifunctional activity. These findings provide a model for how viruses coordinately regulate distinct host defense systems to optimize infection.

## Graphical abstract

Understanding the virus‐host arms race has been a fascinating topic in virology; contributing significantly to the development of treatments and control strategies for viral diseases. CHV1 employs a sophisticated counter‐defense mechanism in its fungal host by utilizing a viral silencing suppressor to inhibit fungal FMN reductase; thereby modulating ROS levels. This alteration triggers autophagic degradation of DCL and AGL proteins; ultimately attenuating the antiviral RNA interference (RNAi) responses.

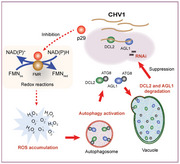

## 1 Introduction

During infection, viruses face a multitude of host antiviral defenses that operate to limit their replication, spread, and pathogenesis; thus, viruses must actively manipulate host physiology to overcome these defenses.^[^ ^1^ ^]^ Reactive oxygen species (ROS), collectively including singlet oxygen (^1^O_2_), superoxide radical (O_2_^•−^), hydroxyl radical (OH^•^), and hydrogen peroxide (H_2_O_2_), serve as important cell signaling molecules for regulating growth and development as well as responses to biotic or abiotic stresses, although in extreme quantities, they may have toxic effects (oxidative stress) on the cell.^[^ ^2^ ^]^ To maintain redox homeostasis and minimize oxidative stress, cells have evolved antioxidative mechanisms involving various ROS‐scavenging enzymes and antioxidant molecules.^[^ ^3^ ^]^ Virus infection often triggers an increased level of ROS (oxidative burst), which has been linked to defense mechanisms like programmed cell death.^[^ ^4^, ^5^ ^]^ Nevertheless, growing evidence suggests that ROS production also benefits viral infection.^[^ ^6^, ^7^, ^8^, ^9^ ^]^ Despite these findings, the precise mechanisms by which viruses exploit ROS signaling to enhance infection remain poorly understood for many viral pathogens.

Autophagy (referring here to macroautophagy) is a cellular process that functions in the degradation or recycling of cellular components and has been implicated in stress responses and antiviral defenses.^[^ ^10^, ^11^ ^]^ In the autophagic pathway, targeted components (cargoes), such as proteins, are recruited into double‐membrane vesicles called autophagosomes, which then transport them to lysosomes (in animals) or vacuoles (in plants and fungi) for hydrolytic degradation.^[^ ^12^ ^]^ Autophagy‐related (ATG) 8 family proteins play central roles in both autophagosome formation and the recruitment of receptors or cargoes.^[^ ^13^, ^14^ ^]^ By targeting viral components for degradation, autophagy restricts viral replication; however, viruses can subvert autophagy.^[^ ^15^, ^16^, ^17^, ^18^, ^19^ ^]^ On the other hand, many viruses utilize autophagy to facilitate infection.^[^ ^20^, ^21^, ^22^, ^23^, ^24^, ^25^ ^]^ In addition, common observations have shown a strong link between oxidative stress and the induction of autophagy.^[^ ^26^, ^27^ ^]^

RNA silencing or RNA interference (RNAi), apart from its role in cellular gene regulation, stands out as an evolutionarily conserved antiviral mechanism targeting the viral genome for degradation or the inhibition of gene expression.^[^ ^28^, ^29^ ^]^ In antiviral RNA silencing, Dicer or Dicer‐like (DCL) enzymes process virus‐derived double‐stranded (ds) RNAs or highly structured RNAs into small interfering RNA duplexes, which are then incorporated into the Argonaute (AGO) or Argonaute‐like (AGL) protein‐containing effector complex called RNA‐induced silencing complex to facilitate sequence specificity in virus inhibition.^[^ ^30^, ^31^, ^32^ ^]^ To counteract RNA silencing, viruses have evolved diverse strategies, most commonly employing virally encoded RNA silencing suppressors.^[^ ^33^, ^34^ ^]^

While autophagy and RNA silencing represent distinct cellular processes mediated by different protein machineries, emerging evidence reveals intriguing cross‐talk between these pathways. Studies have documented autophagic degradation of RNA silencing components including Dicer and AGO proteins in human HeLa cells, cancer cell lines^[^ ^35^, ^36^, ^37^ ^]^ and *Drosophila* systems.^[^ ^38^, ^39^ ^]^ Notably, plant viruses have evolved sophisticated strategies to exploit this interplay, with viral silencing suppressors or hijacked host proteins mediating the autophagic degradation of key silencing factors (AGOs, SGS3, and RDR6) to facilitate infection.^[^ ^40^, ^41^, ^42^ ^]^ However, whether this viral strategy of autophagy‐mediated silencing suppression extends beyond plant systems remains an open and compelling question in host‐pathogen interactions.

Cryphonectria hypovirus 1 (CHV1, genus *Alphahypovirus*, family *Hypoviridae*) is a well‐studied mycovirus originally isolated from the ascomycete fungus *Cryphonectria parasitica* (Diaporthales, Sordariomycetes) that causes chestnut blight disease.^[^ ^43^ ^]^ CHV1 has a positive‐sense single‐stranded RNA genome, measuring 12.7 kb in length and consisting of two continuous open reading frames (ORFs), ORF A and ORF B. ORF A encodes a polyprotein, p69, which is self‐cleaved into p29 and p40.^[^ ^44^, ^45^ ^]^ CHV1 p29 is a papain‐like protease that associates with the trans‐Golgi network and has multifunctional roles in regulating virus accumulation, viability, transmission, and symptom expression.^[^ ^46^, ^47^, ^48^, ^49^, ^50^, ^51^, ^52^ ^]^ CHV1 p29 inhibits RNA silencing by suppressing the transcriptional upregulation of *DCL2* and *AGL2*, two key antiviral silencing genes in *C. parasitica*.^[^ ^53^, ^54^ ^]^ Although the multifaceted roles of p29 have been uncovered, the molecular mechanism underlying its activity is not fully understood. CHV1 represents one of the most studied mycoviruses, with various aspects of virology, including RNA silencing‐mediated antiviral defenses, being covered.^[^ ^53^, ^54^, ^55^, ^56^, ^57^ ^]^ Thus, aside from being a potential biocontrol agent, CHV1 could also serve as an advantageous model virus to explore different aspects of virus‐host interactions.

To elucidate the molecular mechanisms by which CHV1 p29 modulates fungal physiology, we conducted a systematic screen for host interactor proteins in *F. graminearum*. This approach identified a flavin mononucleotide (FMN) reductase as an interacting partner of p29. Further characterization revealed that this interaction triggers increased ROS accumulation and subsequently induces the autophagic degradation of RNA silencing components. Our findings demonstrate a novel viral strategy wherein CHV1 manipulates host oxidative stress pathways to activate autophagy, thereby counteracting the antiviral RNA silencing machinery.

## 2 Results

### 2.1 CHV1 p29 Interacts with an FMN Reductase and Reduces Its Enzymatic Activities

**Figure 1:**
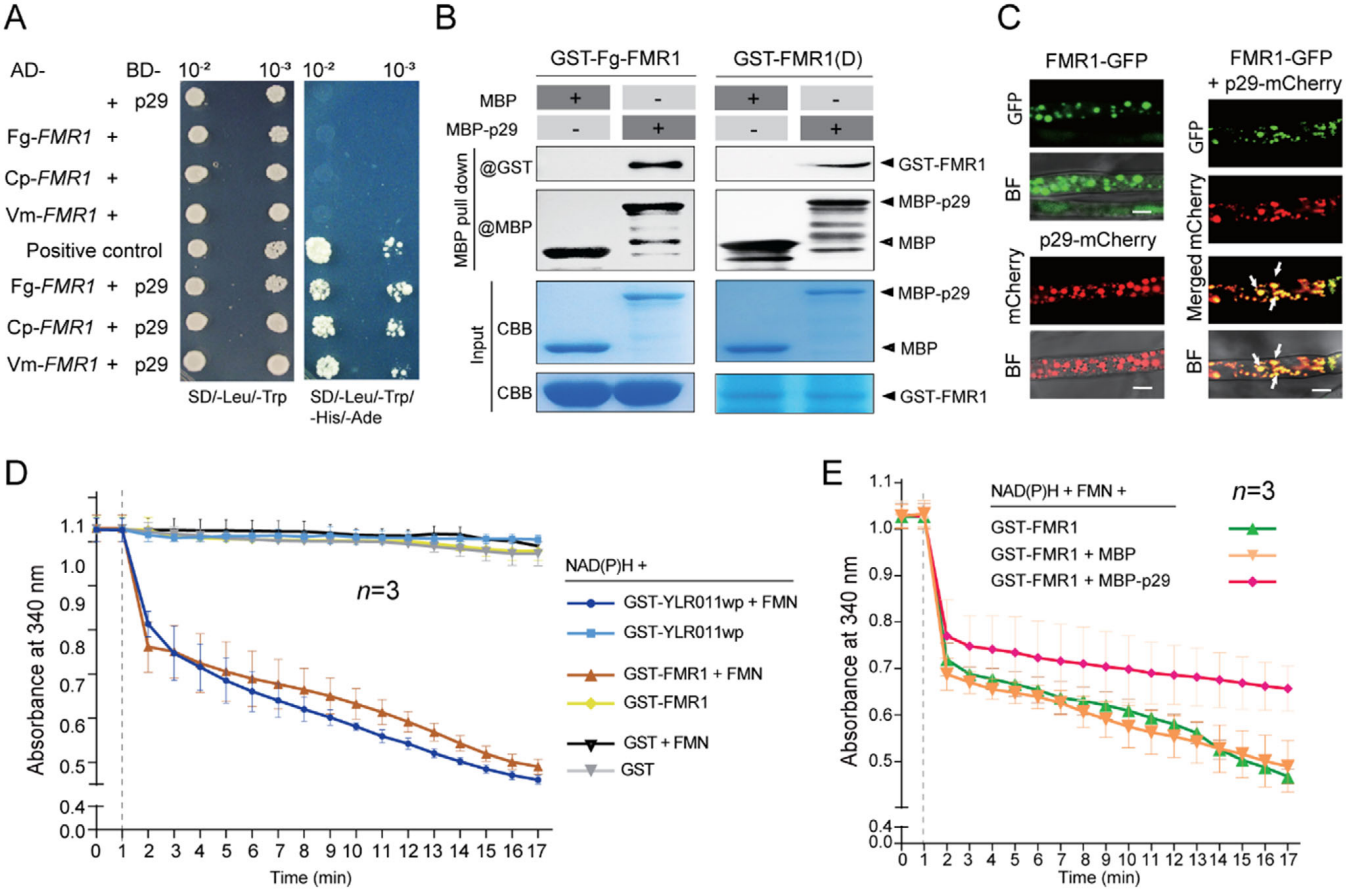
Interaction of p29 with NADPH‐dependent FMN reductase. A) Interaction of p29 with FMR1 in a yeast two‐hybrid assay. FMR1 encoded by *F. graminearum*, *C. parasitica*, and *V. mali* (Fg‐FMR1, Cp‐FMR1, and Vm‐FMR1, respectively) were fused to the Gal4 activation domain (AD), and p29 was fused to the Gal4 DNA‐binding domain (BD). Yeast transformants harboring constructs were grown on selective synthetic defined (SD) media lacking leucine (‐Leu), tryptophan (‐Trp), histidine (‐His), and adenine (‐Ade). Dilution (10‐fold) of yeast cultures is indicated above the panel. AD‐T and BD‐p53 are used as positive control. B) Interaction of p29 with *F. graminearum* FMR1 or FMR1 domain (D) via an in vitro pull‐down assay. Prokaryotically expressed MBP‐p29 was incubated with GST‐FMR1 and ‐FMR1(D), after which MBP pull‐down was performed. Eluted protein samples after the MBP pull‐down were subjected to immunoblotting with anti‐MBP and anti‐GST antibodies. Expressed fusion proteins were run on sodium dodecyl sulfate‐polyacrylamide gel electrophoresis (SDS‐PAGE) and stained with Coomassie brilliant blue (CBB). C) Subcellular colocalization of FMR1‐GFP and p29‐mCherry in *F. graminearum*. Fluorescence signals in mycelial cells were observed by confocal laser scanning microscopy. Scale bar, 5 µm. D) NAD(P)H‐dependent FMN reductase activity of FMR1. GST‐FMR1 was incubated with NAD(P)H and then after the addition of FMN, the oxidation of NAD(P)H was monitored by measuring the decrease in absorption at 340 nm. Yeast YLR011wp, an enzyme with NAD(P)H‐dependent FMN reductase activity was included as a control. E) Effect of p29 on FMR1 enzymatic activity. GST‐FMR1 was mixed with MBP‐p29 or unfused MBP and then incubated with NAD(P)H and FMN. The error bars represent the mean ± standard deviation (SD) of three biological replicates.

Our previous study showed the compatibility of *Fusarium graminearum* as a host of CHV1.^[^ ^58^ ^]^ To screen for host proteins that interact with p29, an in vitro maltose‐binding protein (MBP) pull‐down was carried out using prokaryotically expressed MBP‐p29 incubated with the total proteins extracted from the mycelia of *F. graminearum*. The p29‐binding protein fractions were then subjected to liquid chromatography with tandem mass spectrometry (LC‐MS‐MS) analysis. Several candidate p29‐interacting proteins were identified (Table S1, Supporting Information). A protein (Acc. No. XP_01 132 2075.1) containing an NAD(P)H‐dependent FMN reductase domain, hereafter referred to as FMN reductase 1 (FMR1), was identified among the interacting protein candidates (Figure S1A, Supporting Information) and selected for further analyses. This protein was of particular interest because multiple studies have implicated certain FMN reductases in mitigating oxidative stress (see Discussion section). FMN reductase (oxidoreductase) belongs to a group of flavin‐dependent enzymes, generally called flavoproteins, which utilize flavin derivatives, most commonly FMN and flavin adenine dinucleotide, as cofactors to catalyze redox and non‐redox reactions involved in various metabolic pathways in all organisms.^[^ ^59^ ^]^ By utilizing NADH or NAD(P)H as cofactors, FMN reductase reduces FMN through two‐electron transfer from NADH or NAD(P)H.^[^ ^60^ ^]^ Flavoproteins are particularly notable for various biotechnological and pharmaceutical applications because these enzymes use a wide range of compounds as substrates to produce catalytic products.^[^ ^61^ ^]^ Based on BLAST results, FMR1 had no identity to any well‐characterized flavoproteins, but a conserved domain database search (NCBI) revealed that FMR1 contains the FMN reductase SsuE superfamily domain (Figure S1A, Supporting Information), which is present in the bacterial flavin‐dependent two‐component monooxygenase system.^[^ ^62^ ^]^ Unlike the conventional flavoprotein (flavin reductase), in which the catalytic cycle consists of both a reductive half‐reaction and an oxidative half‐reaction within the same active site, this two‐component system contains separate flavin reductase and monooxygenase enzymes to carry out the reductive and oxidative half‐reactions; reductase produces reduced flavin, which is then used by monooxygenase for molecular oxygen activation.^[^ ^63^, ^64^ ^]^ So far, the presence of this two‐component monooxygenase system has not been reported in higher organisms; thus, it is not clear whether FMR1 is a component of such a system. It was noted that the predicted molecular weight of FMR1 (≈22 kDa) is close to that of the bacterial SsuE protein (≈21 kDa), which is much lower than the molecular weight of conventional flavin reductases (36−49 kDa) identified from *F. graminearum*.^[^ ^65^ ^]^ FMR1 shared moderate amino acid sequence identities, particularly in the domain region, with the homologs encoded by *C. parasitica* and *Valsa mali* (Cp‐FMR1 and Vm‐FMR1, respectively, Figure S1B, Supporting Information). The complete coding sequences of the *FMR1* genes of these three fungal species were cloned, and their interactions with p29 were tested using yeast two‐hybrid and in vitro MBP pull‐down assays. Both assays indicated p29 interaction with the three FMN reductase proteins (**Figure** **1**A,B; Figure S2, Supporting Information). In addition, the MBP pull‐down assay showed p29 interaction with the FMN reductase domain region of FMR1 (Figure 1B).

We then examined the subcellular distribution of FMR1 and p29 by expressing green fluorescent protein (GFP)‐tagged FMR1 (FMR1‐GFP) and monomeric cherry red fluorescent protein (mCherry)‐tagged p29 (p29‐mCherry) in *F. graminearum* and measuring the fluorescence signals using confocal microscopy. When expressed alone, FMR1‐GFP and p29‐mCherry similarly localized in numerous granular structures in the cytosol (Figure 1C), suggesting that these two proteins associate with membranous structures. This observation is in accordance with previous results showing the association of p29 with the trans‐Golgi network.^[^ ^51^ ^]^ Moreover, oxidoreductases are known to commonly associate with endomembranes.^[^ ^66^, ^67^ ^]^ When co‐expressed, FMR1‐GFP and p29‐mCherry evidently co‐localized in granular and aggregate structures (Figure 1C), suggesting their potential association in vivo.

Next, the NAD(P)H‐dependent reductase activity of FMR1 was assayed in the presence or absence of p29. A biochemical reaction was performed using prokaryotically expressed MBP‐p29 and glutathione S‐transferase (GST)‐FMR1 with the addition of NAD(P)H and FMN as an electron donor and a cofactor, respectively, following which the oxidation of NAD(P)H was monitored by measuring the decrease in absorption at 340 nm. Yeast YLR011wp, an enzyme with NAD(P)H‐dependent FMN reductase activity^[^ ^68^ ^]^ was used as a positive control. In the presence of FMN, the rate of NAD(P)H oxidation by FMR1 was comparable to that of YLR011wp, whereas in the absence of FMN, NAD(P)H oxidation did not occur (Figure 1D), indicating that FMR1 is a FMN‐dependent enzyme. The addition of MBP‐p29, but not unfused MBP, resulted in a markedly higher level of absorption (Figure 1E), indicating decreased NAD(P)H oxidation. Thus, the binding of p29 to FMR1 appears to interfere with the enzymatic activity of FMR1.

### 2.2 FMR1 Contributes to Fungal Virulence and Resistance Against CHV1

**Figure 2:**
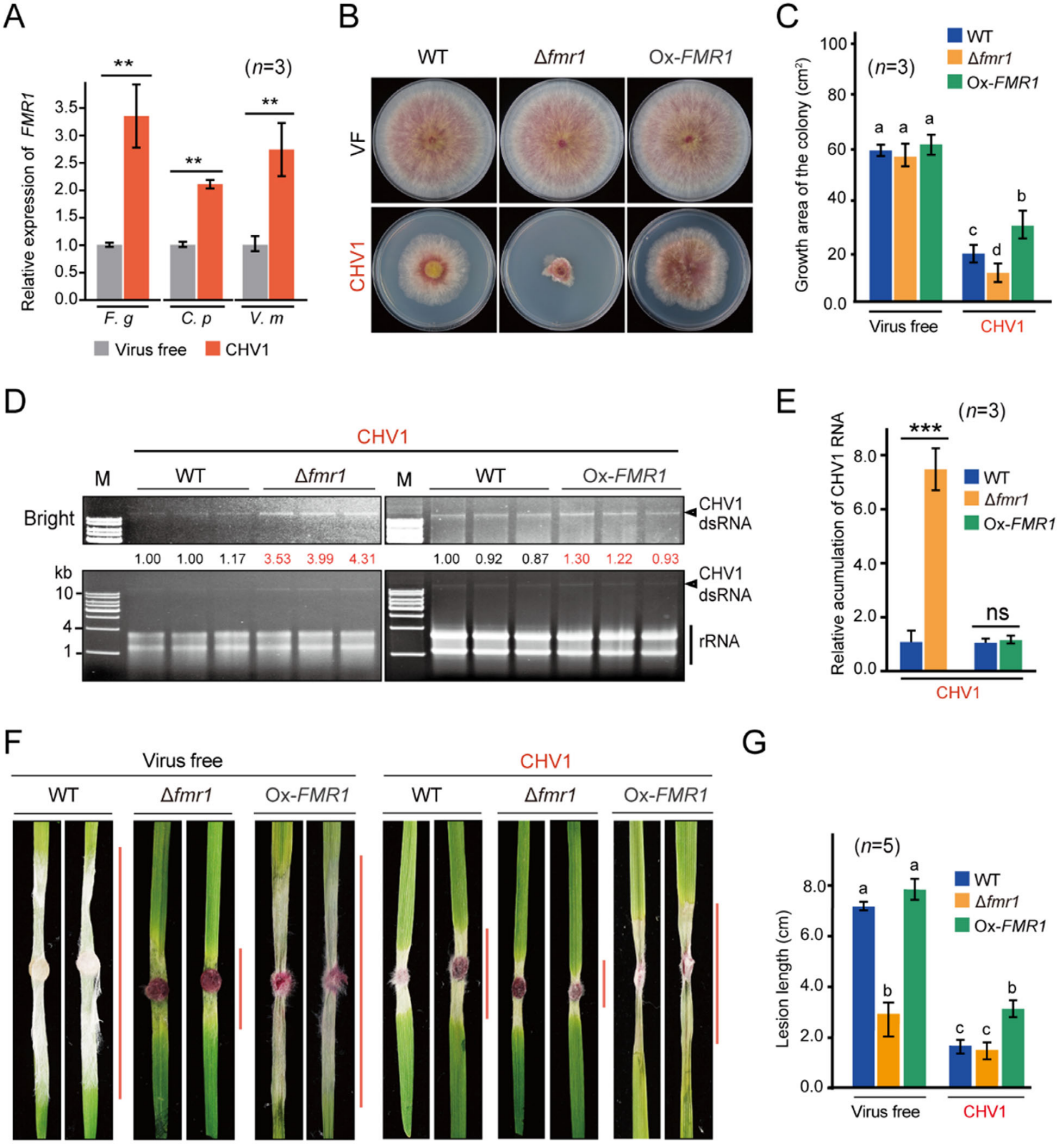
Contribution of FMR1 to fungal virulence and resistance against CHV1. A) Relative transcript levels of *FMR1* in *F. graminearum* (*F. g*), *C. parasitica* (*C. p*), and *V. mali* (*V. m*) upon CHV1 infection analyzed by qRT‐PCR. The virus‐free sample was set to a value of 1.0. Error bars indicate the mean ± standard deviation (SD) of three biological replicates. “**” indicates significant differences at *P* < 0.01 and “ns” indicates nonsignificant differences (Student's *t*‐test). B) Colony growth and morphology of *F. graminearum* wild‐type (WT), *FMR1* knockout (Δ*fmr1)*, and *FMR1*‐overexpressing (Ox‐*FMR1)* strains with or without CHV1 infection grown on PDA medium. Fungal colonies were photographed 3 days after culturing. C) Fungal colony sizes observed in the experiment described in B. Error bars indicate the mean ± standard deviation (SD) of three biological replicates. Different letters indicate significant differences (*P* < 0.05, one‐way ANOVA, Tukey's HSD test). D) CHV1 dsRNA accumulation in Δ*fmr1* and Ox‐*FgFMR1* strains examined by gel electrophoresis of total RNA. The numbers below the dsRNA panel represent the relative signal levels quantified using ImageJ software (National Institutes of Health). The numbers were calculated after normalization with the signal levels of rRNA, and the value from the WT sample was set to 1.00. E) CHV1 RNA accumulation levels in Δ*fmr1* and Ox‐*FMR1* strains analyzed by qRT‐PCR. Error bars indicate the mean ± standard deviation (SD) of three biological replicates. “***” indicates significant differences at *P* < 0.001 and “ns” indicates nonsignificant differences (Student's *t*‐test). F) Inoculation assay of WT, Δ*fmr1*, and Ox‐*FMR1* strains with or without CHV1 infection on wheat leaves. Vertical red lines indicate the fungal lesion area. Photographs were taken 5 days after inoculation. G) Fungal lesion lengths observed in the experiment described in F. The error bars represent the mean ± SD of five biological replicates. Different letters indicate significant differences (*P* < 0.05, one‐way ANOVA, Tukey's HSD test).

Quantitative real‐time PCR (qRT‐PCR) showed that CHV1 infection upregulated the transcriptional expression of the *FMR1* gene in *C. parasitica*, *F. graminearum*, and *V. mali* (**Figure** **2**A), suggesting the possibility that FMR1 is implicated in host defense responses against CHV1 infection. To assess FMR1 function, *FMR1‐*disrupted mutant and overexpressing strains (Δ*fmr1* and Ox‐*FMR1*, respectively) were generated from *F. graminearum* (Figure S3A–D, Supporting Information). CHV1 was then introduced to Δ*fmr1* and Ox‐*FMR1* through hyphal fusion (Figure S3E). Virus‐free Δ*fmr1* and Ox*FMR1* showed similar growth and phenotypes to the wild‐type (WT) strain on potato dextrose agar (PDA) medium. However, when infected with CHV1, Δ*fmr1* exhibited much more reduced growth, whereas Ox‐*FMR1* showed greater growth compared to the WT strain (Figure 2B,C). Analyses of CHV1 dsRNA accumulation levels using gel electrophoresis and RNA accumulation levels using qRT‐PCR showed that the disruption of *FMR1* enhanced CHV1 accumulation, while the overexpression of FMR1 had no effect on CHV1 accumulation (Figure 2D,E). These results indicate that FMR1 is critical for suppressing CHV1 accumulation in *F. graminearum*. Moreover, additional FMR1 expression did not further enhance CHV1 suppression but rather improved fungal stress tolerance against CHV1 infection.

To assess fungal pathogenicity, mycelia were inoculated on wheat leaves, after which fungal lesions were measured. Interestingly, Δ*fmr1* but not Ox‐*FMR1* showed significantly reduced lesion sizes compared to the WT strain (Figure 2F,G). When infected with CHV1, Δ*fmr1* and the WT strain had similar lesion sizes, while Ox‐*FMR1* developed larger lesions (Figure 2F,G). Likewise, Δ*fmr1* showed reduced pathogenicity when inoculated on wheat heads (Figure S4, Supporting Information). Taken together, these results indicate that FMR1 is important for fungal virulence and resistance/tolerance to CHV1 infection.

### 2.3 *FMR1* Deletion or p29 Expression Induces Oxidative Stress

**Figure 3:**
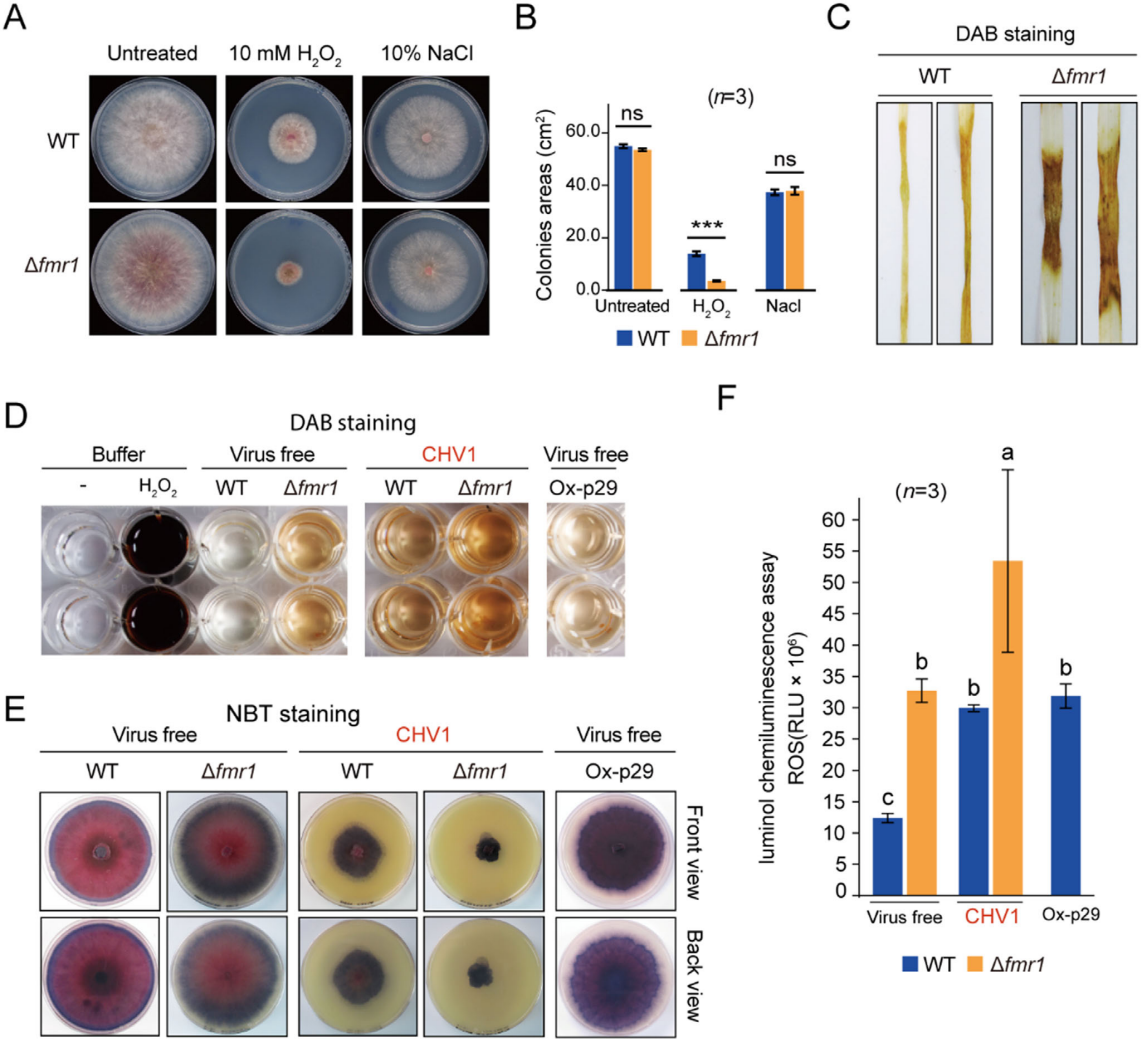
Induction of oxidative stress by *FMR1* deletion or p29 expression. A) Colony growth of *F. graminearum* WT and Δ*fmr1* strains cultured on hyperosmotic (10% NaCl) or oxidative stress‐inducing (10 mM H_2_O_2_) medium. Fungal colonies were photographed 3 days after culturing. B) Fungal colony sizes observed in the experiment described in A. Error bars indicate the mean ± standard deviation (SD) of three biological replicates. “***” indicates significant differences at *P* < 0.001 and “ns” indicates nonsignificant differences (Student's *t*‐test). C) Staining of fungal lesions on wheat leaves using DAB to visualize H_2_O_2_ accumulation. Staining was done 3 days after inoculation. D) DAB staining of fungal mycelia grown on PDA. Staining was done 3 days after culturing. E) Staining of fungal mycelia grown on PDA using NBT to visualize O_2_^•−^ accumulation. Staining was done 3 days after culturing. The experiments were repeated three times, with three replicates each time. F) Chemiluminescent assay to detect H_2_O_2_ accumulation in fungal strains. The error bars represent the mean ± SD of three biological replicates. Different letters indicate significant differences (*P* < 0.05, one‐way ANOVA, Tukey's HSD test).

To further investigate FMR1 function, particularly in protection against abiotic stresses, WT and Δ*fmr1* strains were cultured on media containing 10 mM H_2_O_2_ and 10% NaCl to induce oxidative stresses and hyperosmotic, respectively. While both strains showed similarly reduced growth rates under osmotic stress conditions, the growth of the Δ*fmr1* strain was much more restricted than that of the WT strain on oxidative stress‐inducing medium (**Figure** **3**A,B). This observation suggests that FMR1 contributes to fungal tolerance for oxidative stress. We then examined ROS levels (H_2_O_2_) in fungal strains during infection of wheat leaves via 3‐3′ diaminobenzidine (DAB) staining. The results revealed a marked increase in ROS accumulation in Δ*fmr1*, as indicated by the darker staining of fungal lesions relative to WT lesions (Figure 3C). We then carried out DAB staining and chemiluminescent assay to detect H_2_O_2_ accumulation and nitroblue tetrazolium (NBT) staining to detect O_2_^•−^ accumulation using fungal mycelia. All assays showed an increase in O_2_^•−^ and H_2_O_2_ accumulation, as indicated by darker staining or increased luminol chemiluminescence, in Δ*fmr1* and CHV1‐infected WT and Δ*fmr1* strains relative to the virus‐free WT strain (Figure 3D–F), indicating that *FMR1* deletion and CHV1 infection elevate ROS levels. To examine whether p29 is responsible for increased ROS accumulation upon CHV1 infection, an *F. graminearum* strain expressing p29 (Ox‐p29) was generated and subjected to ROS detection. DAB/NBT staining and a chemiluminescent assay indicated increased ROS accumulation in p29‐expressing strains (Figure 3D–F). This result suggests that p29 expression induces oxidative stress, likely through its interaction with FMR1.

### 2.4 Increased ROS Levels are Correlated with Enhanced CHV1 Accumulation and Autophagic Activity

**Figure 4:**
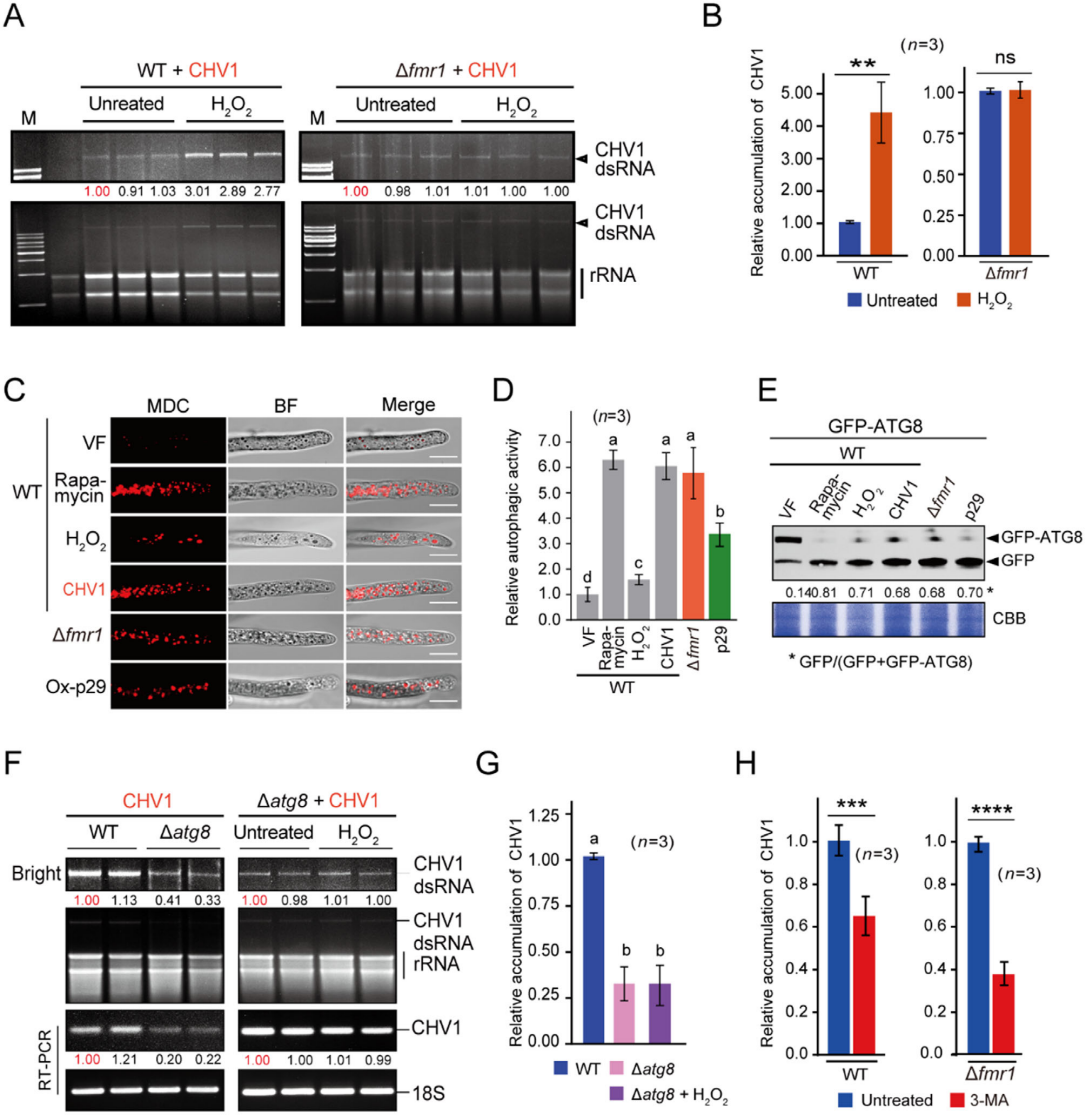
Association of oxidative stress with increased CHV1 accumulation and autophagic activity. A) CHV1 dsRNA accumulation in *F. graminearum* WT and Δ*fmr1* strains grown on oxidative stress‐inducing medium. B) CHV1 RNA accumulation levels in fungal strains described in A analyzed by qRT‐PCR. The untreated sample value was set to 1.0. “**” indicates significant differences at *P* < 0.01 and “ns” indicates nonsignificant differences (Student's *t*‐test). C) Staining of autophagosomes in the mycelia of *F. graminearum* WT, Δ*fmr1*, and Ox‐p29 strains using the fluorescent dye MDC. Fluorescence signals in mycelial cells were observed by confocal laser scanning microscopy. Scale bar, 10 µm. D) Relative autophagic activity based on the number of MDC‐stained bodies observed in the experiment described in C. Each bar represents the mean number of structures counted from 100 cells obtained from three independent experiments. The WT virus‐free (VF) sample value was set to 1.0. Different letters indicate significant differences (*P* < 0.05, one‐way ANOVA, Tukey's HSD test). E) GFP‐ATG8 processing assay to monitor autophagic flux in fungal strains. GFP‐ATG8 was expressed in fungal strains and subjected to western blotting using an anti‐GFP antibody. Detection of free GFP indicates GFP‐ATG8 processing in the vacuole. F) CHV1 dsRNA accumulation in *F. graminearum* WT and *ATG8* knockout (*Δatg8)* strains grown on untreated or oxidative stress‐inducing medium. G) CHV1 RNA accumulation levels in fungal strains described in F analyzed by qRT‐PCR. The WT untreated sample value was set to 1.0. Different letters indicate significant differences (*P* < 0.05, one‐way ANOVA, Tukey's HSD test). H) CHV1 RNA accumulation levels in *F. graminearum* WT and Δ*fmr1* strains treated with the autophagy inhibitor 3‐MA analyzed by qRT‐PCR. The untreated sample value was set to 1.0. “***” “****” indicates significant differences at *P* < 0.001 and < 0.0001, respectively and “ns” indicates nonsignificant differences (Student's *t*‐test). The error bars represent the mean ± SD of three biological replicates.

As *FMR1* deletion was correlated with increased CHV1 accumulation and ROS levels (Figure 2D–E and Figure 3C,D), we questioned whether oxidative stress per se could enhance CHV1 accumulation. To this end, a CHV1‐infected WT strain was cultured on media containing H_2_O_2,_ following which virus accumulation was examined. The accumulation of CHV1 RNAs was remarkably higher in H_2_O_2_‐containing media than in untreated media (**Figure** **4**A,B). This indicates that increased ROS levels positively affect CHV1 accumulation. When a CHV1‐infected Δ*fmr1* strain was cultured on H_2_O_2_‐containing media, the CHV1 dsRNA accumulation level was similar to that on untreated media (Figure 4A,B), indicating that *FMR1* deletion and H_2_O_2_ treatment have no redundant effect on CHV1 accumulation.

Considering the established link between ROS and autophagy, particularly in the context of autophagy activation by oxidative stress,^[^ ^26^ ^]^ we then examined whether the induction of oxidative stress by H_2_O_2_ treatment, *FMR1* deletion, CHV1 infection, or p29 expression could activate autophagy in *F. graminearum*. Autophagy activation in the cells was observed using a fluorescent dye with monodansylcadaverine (MDC) that stains autophagosomes.^[^ ^69^ ^]^ Under confocal microscopy, increased numbers of autophagosomes in fungal cells were observed under all the tested conditions as well as treatment with the autophagy inducer rapamycin, with the largest increase observed for CHV1 infection, *FMR1* deletion and rapamycin treatment (Figure 4C,D). Next, we performed a GFP‐ATG8 processing assay to monitor autophagic flux.^[^ ^70^ ^]^ In this assay, the increase of free GFP indicates GFP‐ATG8 processing in the vacuole. This assay further confirmed autophagy activation upon H_2_O_2_ treatment, CHV1 infection, p29 expression, or *FMR1* deletion (Figure 4E). These observations suggest that an increase in ROS levels is correlated with autophagy activation in *F. graminearum*.

As increased ROS levels are also associated with higher levels of CHV1 accumulation, autophagy seems to promote CHV1 accumulation, as observed for many viruses. A previous study showed that the deletion of *ATG8* in *C. parasitica* reduced CHV1 accumulation,^[^ ^71^ ^]^ indicating that autophagy is required for optimal CHV1 accumulation. To determine whether autophagy in CHV1 infection also plays such a proviral role in *F. graminearum*, an *ATG8*‐disrupted mutant strain of *F. graminearum* (Δ*atg8*) was generated and inoculated with CHV1. In the absence of CHV1 infection, the Δ*atg8* strain exhibited reduced growth compared to the wild‐type strain, demonstrating ATG8's importance for normal fungal growth. Strikingly, while CHV1 infection significantly reduced wild‐type strain growth, it showed no observable effect on Δ*atg8* growth (Figure S5, Supporting Information). As expected, CHV1 RNA accumulation was reduced in the Δ*atg8* strain, but the low CHV1 RNA accumulation in the Δ*atg8* strain could not be enhanced with H_2_O_2_ treatment (Figure 4F,G), suggesting that autophagy is involved in the H_2_O_2_‐mediated enhancement of CHV1 accumulation. Moreover, treatment with the autophagy inhibitor 3‐methyladenine (3‐MA) reduced CHV1 accumulation in the WT and the Δ*fmr1* strains (Figure 4H), confirming the positive role of autophagy in optimal infection by CHV1.

To further validate the key role of ROS in enhanced viral accumulation and autophagy activation following *FMR1* deletion, the CHV1‐infected *Δfmr1* strain was treated with the ROS inhibitor N‐acetyl‐L‐cysteine (NAC). Consistent with this view, NAC treatment reduced ROS levels, which correlated with decreased CHV1 accumulation and fewer autophagosomes in the Δ*fmr1* strain (Figure S6, Supporting Information).

### 2.5 DCL2 and AGL1 are Key Antiviral Components Against CHV1 and Interact with ATG8

The role of autophagy in promoting virus infection can be ascribed to the ability of viruses to hijack autophagic machinery and directly utilize it for specific processes in their life cycle, such as replication, assembly, maturation, and cell‐to‐cell spread.^[^ ^72^ ^]^ Alternatively, viruses can exploit autophagy to suppress host antiviral responses.^[^ ^73^, ^74^ ^]^ Notably, as mentioned in the introduction, several plant viruses were shown to utilize autophagic pathways to degrade RNA silencing components; however, it is unknown whether non‐plant viruses have also evolved an ability to exploit autophagy in this manner. Given that RNA silencing is a major antiviral strategy in fungi, we were prompted to investigate whether the proviral roles of autophagy in CHV1 infection are related to the suppression of fungal antiviral RNA silencing.

**Figure 5:**
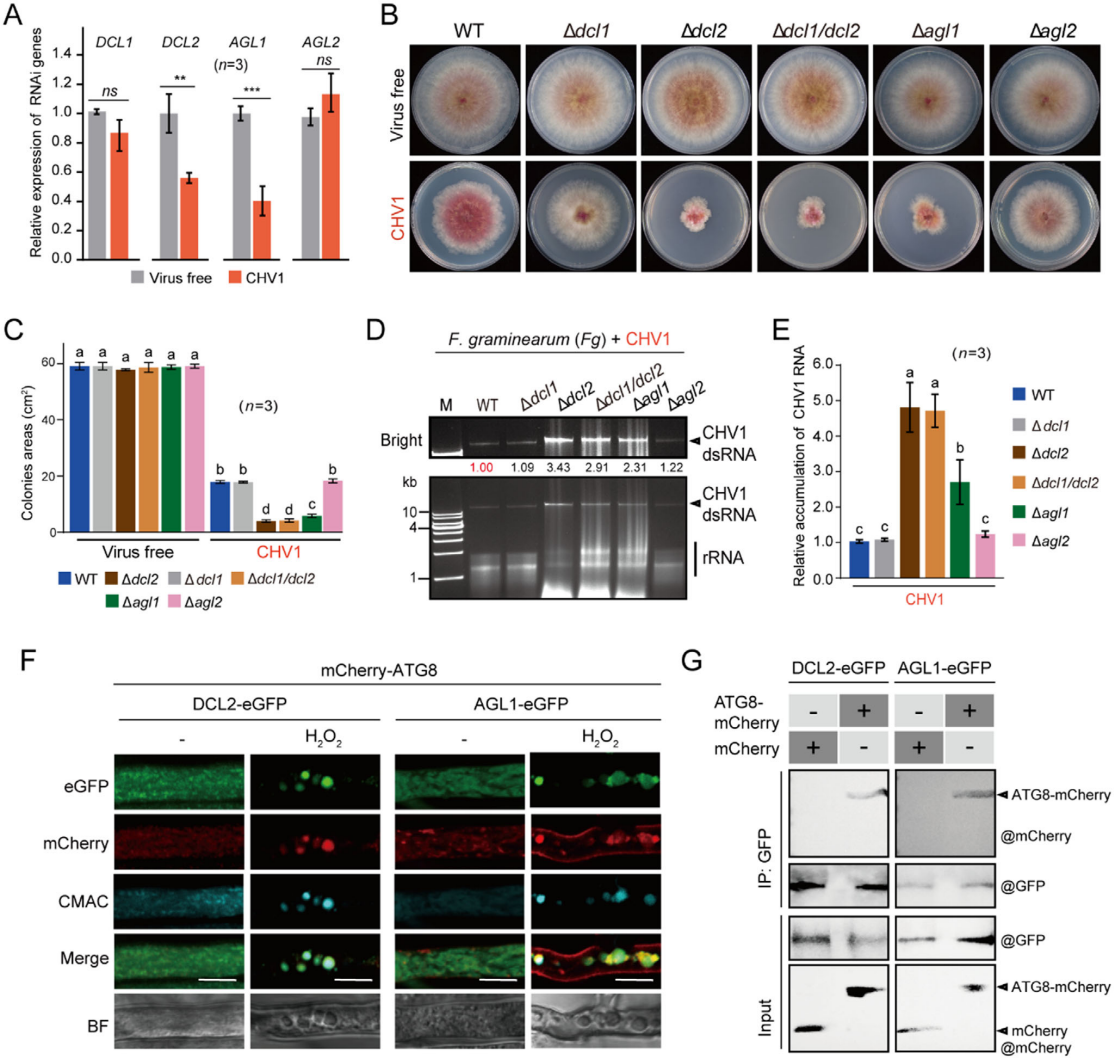
Antiviral roles of DCL2 and AGL1 and their interaction with ATG8. A) Relative transcript levels of *DCL* and *AGL* genes in *F. graminearum* upon CHV1 infection analyzed by qRT‐PCR. The virus‐free sample was set to a value of 1.0. “**” “***” indicates significant differences at *P* < 0.01 and < 0.001, respectively and “ns” indicates nonsignificant differences (Student's *t*‐test). B) Colony growth and morphology of *F. graminearum* WT, *DCL*, and *AGL* mutant strains with or without CHV1 infection grown on PDA medium. Fungal colonies were photographed 3 days after culturing. C) Fungal colony sizes observed in the experiment described in B. Different letters indicate significant differences (*P* < 0.05, one‐way ANOVA, Tukey's HSD test). D) CHV1 dsRNA accumulation levels in WT and knockout mutant strains. E) CHV1 RNA accumulation in the fungal strains described in C analyzed by qRT‐PCR. The WT sample value was set to 1.0. Different letters indicate significant differences (*P* < 0.05, one‐way ANOVA, Tukey's HSD test). The error bars represent the mean ± SD of three biological replicates. F) Subcellular colocalization of mCherry‐ATG8 and DCL2‐eGFP or AGL1‐eGFP in *F. graminearum*. Scale bar, 10 µm. G) Interaction of ATG8‐mCherry and DCL2‐eGFP or AGL1‐eGFP in a pull‐down assay. Protein samples before and after the GFP pull‐downs were subjected to immunoblotting with anti‐GFP and anti‐mCherry antibodies.

First, we identified the key DCL and AGL components of antiviral silencing against CHV1 in *F. graminearum*. Two *DCL* genes (*DCL1* and *DCL2*) and two *AGL* genes (*AGL1* and *AGL2*) are present in the *F. graminearum* genome.^[^ ^75^ ^]^ CHV1 infection downregulated the transcriptional expression of *DCL2* and *AGL1* but did not affect *DCL1* and *AGL2* expressions (**Figure** **5**A). CHV1 was introduced to each single disruption mutant of *DCL1*, *DCL2*, *AGL1*, and *AGL2*, as well as the double disruption mutant of *DCL1* and *DCL2*, following which virus accumulation was analyzed. The Δ*dcl1*, Δ*dcl2*, and Δ*dcl1*/*dcl2* strains were generated in a previous study,^[^ ^76^ ^]^ while the Δ*agl1* and Δ*agl2* strains were generated in this study (Figure S7, Supporting Information). While all virus‐free mutant strains exhibited similar phenotypic growth on PDA medium, CHV1 infection particularly caused more severe growth inhibition in the Δ*dcl2*, Δ*dcl1*/*dcl2*, and Δ*agl1* mutant strains (Figure 5B,C). CHV1 accumulation was significantly increased in Δ*dcl2*, Δ*dcl1*/*dcl2*, and Δ*agl1* (Figure 5D,E). These results indicate that DCL2 and AGL1 are the key components of antiviral RNA silencing against CHV1 in *F. graminearum*. Thus, although CHV1 infection downregulates their expression, this suppression does not completely abolish their antiviral activity.

Next, we investigated whether DCL2 and AGL1 are affected by autophagy. DCL2 and AGL1 were fused with eGFP (DCL2‐eGFP and AGL1‐eGFP, respectively) and expressed in *F. graminearum* (Figure S8A, Supporting Information) together with ATG8, which is widely used as an autophagosome marker.^[^ ^77^ ^]^ We used an ATG8 homolog in *F. graminearum* ^[^ ^78^ ^]^ tagged with mCherry (mCherry‐ATG8). Confocal microscopic observation of fungal cells showed that DCL2‐eGFP and AGL1‐eGFP similarly distributed throughout the cytoplasm. Interestingly, when the fungi were treated with H_2_O_2_, fluorescent signals became scarce. However, some cells close to the hyphal tip showed DCL2‐eGFP, AGL1‐eGFP, and mCherry‐ATG8 signals localized in vacuoles, as confirmed by vacuole staining using the fluorescent aminopeptidase substrate 7‐amino‐4‐chloromethylcoumarin (CMAC; Figure 5F), showing that DCL2 and AGL1 are transported to vacuoles upon the activation of autophagy by H_2_O_2_ treatment. DCL2 and AGL1 were predicted to contain multiple potential ATG8‐interacting motifs (Figure S8B, Supporting Information), which mediate the binding of ATG8‐family proteins to cargo receptors or substrates.^[^ ^79^ ^]^ To examine whether DCL2 or AGL1 interacts with ATG8, DCL2‐eGFP or AGL1‐eGFP was co‐expressed with ATG8‐mCherry in WT *F. graminearum* (without CHV1 infection or autophagy‐inducing treatment) and then subjected to an in vivo co‐immunoprecipitation (co‐IP) assay using a GFP antibody. The co‐IP assay indicated the binding of ATG8 and DCL2 or AGL1 (Figure 5G).

### 2.6 Increased ROS Levels Induce the Autophagic Degradation of DCL2 and AGL1

**Figure 6:**
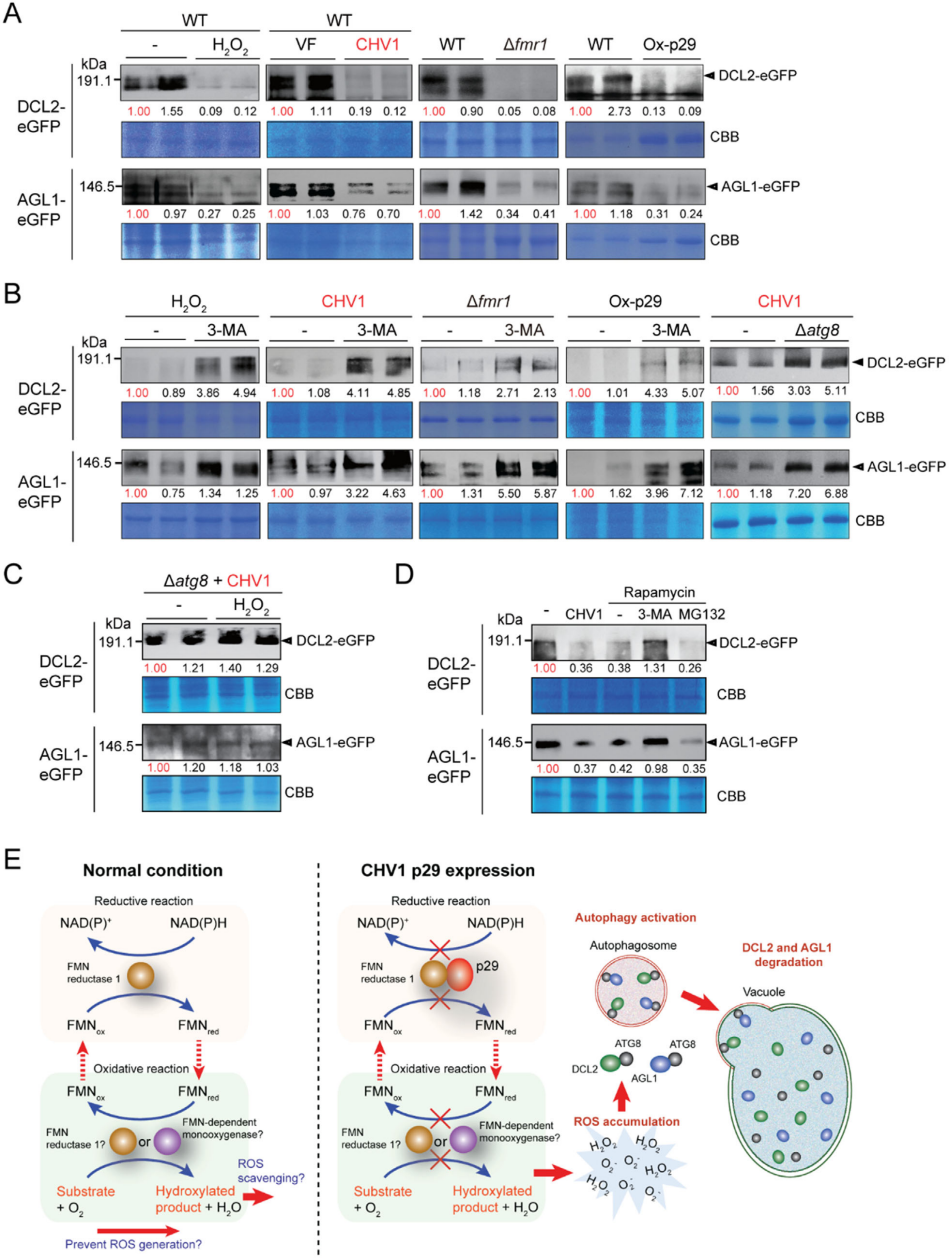
Induction of autophagic degradation of DCL2 and AGL1 by oxidative stress. A) DCL2‐eGFP and AGL1‐eGFP accumulation levels in *F. graminearum* with H_2_O_2_ treatment, CHV1 infection, *FMR1* deletion (Δ*fmn_red_*), or p29 overexpression (Ox‐p29) assessed by western blotting using an anti‐GFP antibody. Sodium dodecyl sulfate‐polyacrylamide gel electrophoresis (SDS‐PAGE) of total protein samples was stained with Coomassie brilliant blue (CBB) and used as a loading control. The numbers below the blots represent the relative signal levels quantified using ImageJ software. The numbers were calculated after normalization with the signal levels of CBB‐stained proteins, and the value from the untreated sample was set to 1.00. B) Restoration of DCL2‐eGFP and AGL1‐eGFP accumulation by treatment with an autophagy inhibitor (3‐MA) or deletion of *ATG8* (Δ*atg8*) assessed by western blotting. C) DCL2‐eGFP and AGL1‐eGFP accumulation levels in CHV1‐infected Δ*atg8* with or without H_2_O_2_ treatment assessed by western blotting. D) Induction of DCL2‐eGFP and AGL1‐eGFP degradation by an autophagy inducer (rapamycin) assessed by western blotting. 3‐MA or proteasome inhibitor MG132 was added to verify that degradation is due to autophagy, not proteasomal degradation. E) A model for the induction of DCL2 and AGL1 autophagic degradation through the inhibition of FMN reductase activity by CHV1 p29. In normal conditions, FMR1 may carry out both the reductive half‐reaction and the oxidative half‐reaction within the same active site; alternatively, an unknown monooxygenase enzyme may carry out the oxidative half‐reaction to catalyze the substrates. The catalytic products may contribute to antioxidant activities and/or the reduction of the carbon‐carbon double bond in α/β‐unsaturated substrates, thereby preventing ROS generation. During CHV1 infection, p29 binds and inhibits the activity of FMR1. This results in increased ROS accumulation and subsequently leads to autophagy activation. ATG8 serves as a cargo receptor to recruit DCL2 and AGL1 into autophagosomes through direct interaction. DCL2 and AGL1 are then transported to vacuoles for hydrolytic degradation.

As DCL2 and AGL1 appeared to localize with ATG8 in the vacuoles following the activation of autophagy and were thus likely targeted for degradation, we then analyzed the accumulation of DCL2‐eGFP and AGL1‐eGFP in *F. graminearum* upon H_2_O_2_ treatment, CHV1 infection, p29 expression (Figure S8C, Supporting Information), or *FMR1* deletion by an immunoblot assay using a GFP antibody. The assay showed that all the above conditions markedly reduced DCL2‐eGFP and AGL1‐eGFP accumulation but not that of unfused GFP (**Figure** **6**A; Figure S8D, Supporting Information). To verify that the reduction of protein accumulation is due to autophagic degradation, fungal strains were then treated with 3‐MA. Treatment with 3‐MA restored DCL2‐eGFP and AGL1‐eGFP accumulation to a certain extent but did not affect free GFP accumulation in fungal strains with H_2_O_2_ treatment, CHV1 infection, p29 expression, or *FMR1* deletion (Figure 6B; Figure S8D, Supporting Information). Moreover, DCL2‐eGFP and AGL1‐eGFP accumulation was also restored in the Δ*atg8* strain infected with CHV1 (Figure 6B), but DCL2‐eGFP and AGL1‐eGFP accumulation in the Δ*atg8* strain were not affected by H_2_O_2_ treatment (Figure 6C), further confirming the role of autophagy in H_2_O_2_‐induced DCL2 and AGL1 degradation. In further experiments, when fungal strains were treated with the autophagy inducer rapamycin, DCL2‐eGFP and AGL1‐eGFP accumulation was reduced, and this reduction could be blocked by 3‐MA but not the proteasome inhibitor carbobenzoxy‐leucyl‐leucyl‐leucinal (MG132) (Figure 6D). RT‐PCR analysis indicated that the change in protein accumulation presented in Figure 6 was not due to changes in transcript levels (Figure S9, Supporting Information). Taken together, these observations confirmed that the autophagy pathway is responsible for DCL2‐eGFP and AGL1‐eGFP degradation during oxidative stress.

## 3 Discussion

While CHV1 p29 is recognized as a multifunctional protein critical for viral infection and pathogenesis, its mechanisms of action in modulating fungal metabolism and physiology remain poorly understood. Notably, no host interaction partners of p29 had been previously identified. Building on our earlier discovery that *F. graminearum* serves as a compatible host for CHV1, where the virus induces hypovirulence and p29 remains functional,^[^ ^58^, ^80^ ^]^ we leveraged this system to investigate virus‐host interactions. The extensive genetic tools available for *F. graminearum*, coupled with its well‐characterized physiology, made it an ideal heterologous system for this study. This study revealed that by inhibiting the activity of a host FMN reductase via p29, CHV1 elevates ROS levels to activate autophagy, which in turn targets key RNA silencing components for degradation (Figure 6E). Our results provide novel insights into the crucial connections among ROS, autophagy, and RNA silencing and how the virus can co‐opt these interconnected pathways to promote infection.

The biological function of FMR1 and its homologs in filamentous fungi remains uncharacterized. Our study provides the first demonstration of FMN‐dependent electron transfer activity in FMR1 (Figure 1D). As FMR1 contains an FMN reductase domain belonging to the bacterial flavin‐dependent two‐component monooxygenase system, which is typically a flavin‐free enzyme complex,^[^ ^81^, ^82^ ^]^ it intriguingly seems not to be a canonical flavoprotein, which usually has flavin covalently or non‐covalently bound.^[^ ^59^ ^]^ The precise biochemical nature of FMR1 and its physiological redox substrates warrant further investigation.

It is still unclear how FMR1 inactivation could lead to an increase in the ROS level (Figure 3). A possible explanation is that redox reactions involving FMR1 or its catalytic products may contribute to reducing cellular ROS levels (Figure 6E). As flavoproteins catalyze the reduction of the carbon‐carbon double bond in α/β‐unsaturated substrates,^[^ ^83^ ^]^ which are chemically reactive,^[^ ^84^ ^]^ they may contribute to mitigate ROS accumulation. Studies in various organisms showed the role of certain types of FMN reductases in reducing ROS levels. FerB, an FMN reductase in the bacterium *Paracoccus denitrificans*, which catalyzes the conversion of ubiquinone to ubihydroquinone, was shown to reduce the accumulation levels of exogenously added O_2_^•−^ or H_2_O_2_.^[^ ^85^ ^]^ Flavin reductase 1 in the microaerophilic protist *Trichomonas vaginalis* is involved in antioxidative defense.^[^ ^86^ ^]^ Studies in mammals and yeast indicated that flavin‐dependent quinone reductases play a role in protection against oxidative stress through the two‐electron reduction of quinone to the hydroquinone form so that the generation of the one‐electron reduced semiquinone form, which tends to react with molecular dioxygen to produce O_2_^•−^, is blocked.^[^ ^87^, ^88^ ^]^ OYE2, an FMN reductase in yeast, can lower endogenous ROS levels and enhance resistance to H_2_O_2_‐induced programmed cell death.^[^ ^89^ ^]^ *Triticum aestivum* (wheat) 12‐oxo‐phytodienoic acid reductase 1, belonging to FMN‐dependent oxidoreductases that catalyze the reduction of double bonds in α,β‐unsaturated aldehydes and ketones, was shown to have ROS scavenging activity.^[^ ^90^ ^]^ It is also important to note that the deletion of *FMR1* largely attenuated the pathogenicity of *F. graminearum* (Figure 2F,G; Figure S4, Supporting Information). Thus, oxidative stress appears to weaken *F. graminearum's* ability to infect and/or induce disease in the host plant. This observation indicates that the pathogenicity‐attenuation function of p29 is, in part, related to the induction of ROS through the inhibition of FMR1 activity.

In previous studies using *C. parasitica*, p29's function in the inhibition of antiviral RNA silencing was attributed to its activity in suppressing the induction of *dcl2* and *agl2* transcripts following CHV1 infection.^[^ ^53^, ^54^ ^]^ The transcriptional upregulation of *dcl2* and *agl2* genes is mediated by the SAGA complex, a well‐known transcriptional coactivator; moreover, DCL2 is required for the SAGA‐mediated upregulation of genes involved in host tolerance against CHV1 infection.^[^ ^56^, ^57^ ^]^ However, it is still unclear how p29 interferes with the SAGA‐mediated induction of *dcl2* and *agl2* transcripts. The results of this study revealed a different aspect of p29 function that operates through the downregulation of protein accumulation of key silencing components via the autophagy pathway. Notably, some RNA silencing suppressors encoded by plant viruses were shown to direct the degradation of RNA silencing components by the autophagy pathway. The P0 protein encoded by poleroviruses acts as an F‐box protein to mediate the ubiquitination of AGO1, leading to autophagic degradation.^[^ ^41^, ^91^, ^92^, ^93^ ^]^ Viral genome‐linked protein (VPg) encoded by turnip mosaic virus interacts with SGS3, and VPg expression induces the degradation of SGS3 and RDR6, its partner in the protein complex, via both the autophagy and the 20S ubiquitin‐proteasome pathways.^[^ ^42^ ^]^ A recent study showed that HC‐Pro, the silencing suppressor encoded by plant viruses in the family *Potyviridae*, interferes with HEN1 methyltransferase activity, leading to autophagic degradation of AGO1.^[^ ^94^ ^]^ A plant virus could utilize host cellular proteins to facilitate the autophagic degradation of SGS3/RDR6 complexes.^[^ ^40^ ^]^ Notably, the DCL degradation induced by viral protein or virus infection has not been reported in plants and animals. Given that DCL proteins play a central role in antiviral RNA silencing, it is worth investigating whether inducing DCL degradation is a general strategy employed by viruses to overcome antiviral responses in various organisms.

Many studies have shown that increased ROS levels have a positive influence on virus replication and pathogenesis, but the molecular mechanism underlying this process is not fully understood for many viruses.^[^ ^6^, ^7^, ^95^ ^]^ CHV1 p29 shares a conserved papain‐like protease domain and sequence similarities with HC‐Pro.^[^ ^96^, ^97^ ^]^ Intriguingly, Chilli veinal mottle virus (ChiVMV) HC‐Pro was shown to interact with and inhibit the enzymatic activity of plant catalases (ROS‐scavenging enzymes), resulting in increased H_2_O_2_ levels and enhanced viral accumulation.^[^ ^98^ ^]^ Another study demonstrated that ChiVMV HC‐Pro suppresses ethylene biosynthesis, which resulted in reduced peroxidase activity and increased ROS accumulation.^[^ ^99^ ^]^ It would be interesting to investigate whether HC‐Pro also modulates ROS levels to subvert autophagy and thereby diminish antiviral defenses. It is also important to note that HC‐Pro has also been identified as a target of autophagy.^[^ ^17^, ^100^ ^]^ Thus, HC‐Pro may function as either a target or an inducer of autophagy, depending on the virus species. The RNA capping activity of animal RNA viruses belonging to the groups alphavirus and flavivirus was shown to be enhanced in oxidative conditions.^[^ ^9^ ^]^ Robust replication of two plant RNA viruses, namely red clover necrotic mosaic virus (genus *Dianthovirus*) and brome mosaic virus (genus *Bromovirus*), was shown to require respiratory burst oxidase homolog B‐derived ROS accumulation.^[^ ^8^ ^]^ Notably, ROS could also indirectly foster virus replication through the modulation of host signaling pathways, as in the case of infection by enterovirus A71 (an RNA virus in the family *Picornaviridae*), which induces endoplasmic reticulum stress/autophagy and activates the NF‐κB and JNK pathways for its robust replication.^[^ ^101^, ^102^, ^103^ ^]^ Likewise, as revealed in this study, ROS appears to indirectly contribute to CHV1 infection through the activation of autophagy, which interferes with the antiviral RNA silencing process. As oxidative stress is commonly associated with the activation of autophagy,^[^ ^26^, ^27^ ^]^ it is interesting to further explore whether viruses generally induce ROS to exploit autophagy pathways to overcome host immunity. Many observations have revealed that autophagy activation following animal virus infection leads to the suppression of host immune responses.^[^ ^72^ ^]^ Considering the trade‐offs between growth/stress tolerance and immunity in living organisms,^[^ ^104^, ^105^, ^106^, ^107^ ^]^ the downregulation of cellular immune pathways by autophagy may be reserved for particular conditions. Thus, by modulating host physiology, viruses take advantage of this cross‐regulation of signaling pathways to overcome defense responses.

## 4 Experimental Section

### Fungal Strains, Viruses, and Fungal Culture

*F. graminearum* strain PH‐1 WT, Δ*dcl1*, Δ*dcl2*, and Δ*dcl1*/Δ*dcl2* mutants^[^ ^76^ ^]^ were kindly provided by Huiquan Liu, Northwest A&F University, China. The *F. graminearum* PH‐1 strain was used as the background for all mutant and transformed strains generated and/or used in this study. The *C. parasitica* strain EP155 virus‐free and CHV1‐infected strains^[^ ^108^ ^]^ were kindly provided by Nobuhiro Suzuki, Okayama University, Japan. The *V. mali* 03–8 strain^[^ ^109^ ^]^ was kindly provided by Lili Huang, Northwest A&F University, China. The introduction of CHV1 to *F. graminearum* PH‐1 has been described previously.^[^ ^58^ ^]^ CHV1 was introduced to the transformed fungal strains by hyphal fusion. All fungal strains were grown on PDA at 25 °C for 3–5 days. For the induction of oxidative stress, 10 mM H_2_O_2_ was added to PDA (8). For nucleic acid extraction, fungi were cultured on PDA layered with a cellophane sheet.

### Total RNA Extraction and Viral dsRNA Analysis

Total RNA was extracted from fungal hyphae using the phenol‐chloroform method described previously.^[^ ^110^ ^]^ Extracted RNA was treated with RQI DNase I, and RNA concentration was adjusted to 2 µg µL^−1^. Total RNA was electrophoresed in 1% agarose gel and stained with ethyl bromide. The relative amount of dsRNA in the virus genome was determined by quantifying the brightness of the RNA band with Image J software (2). The 18S rRNA band was used as the reference standard.

### RT‐PCR and qRT‐PCR

First‐strand cDNA was synthesized using reverse transcriptase (TransGen Biotech, China). PCR amplification was carried out using mixed DNA polymerase (CWBIO, China). qRT‐PCR was performed using KAPA SYBR FAST qPCR Kit Master Mix (2X) ABI Prism (Kapa Biosystems, USA) and run on a CFX96 Touch Real‐Time PCR Detection System (BioRAD, USA). 18S rRNA was used as an internal control. Three biological replicates were used for each sample, and the experiment was repeated three times independently. All primers used in the study were listed in Table S2 (Supporting Information).

### Plasmid Construction and Fungal Transformation

The coding region of genes analyzed in this study was amplified by RT‐PCR. Recombinant plasmids for protein expression in *F. graminearum* were constructed using yeast gap repair method as described previously.^[^ ^111^ ^]^ Plasmids for yeast two‐hybrid assay, and protein expression in *Escherichia coli* were constructed using the Seamless Cloning Kit (Vazyme Biotech, China). All plasmid constructs generated in this study are described in Table S3 (Supporting Information). Deletion (knockout) mutants were generated through homologous recombination using DNA fragments containing a hygromycin expression cassette and target region sequences prepared with double‐joint PCR.^[^ ^112^ ^]^ The recombinant plasmids or DNA fragments were transfected into *F. graminearum* protoplasts by polyethylene glycol‐mediated transformation, as described previously.^[^ ^113^ ^]^ Candidate gene deletion mutants were identified by PCR with specific primers and further analyzed by Southern blotting. At least three gene deletion mutants were obtained.

### MBP Pull‐Down, Yeast Two‐Hybrid, and In Vivo Co‐IP Assays

For the MBP pull‐down assay, FMR1 and p29 fusion proteins were expressed in *E. coli* BL21 and purified. MBP pull‐down was performed according to the previously described method.^[^ ^114^ ^]^ Eluted proteins were analyzed by western blotting. For the yeast two‐hybrid assay, DNA fragments corresponding to *FMR1* and *p29* coding sequences were cloned into yeast two‐hybrid prey/library and bait vectors, respectively (Coolaber Biotech, China). The bait and prey vector constructs were transformed into yeast AH109 in pairs. Transformants were cultured on a selective medium without leucine and tryptophan for 3 days at 30 °C. Yeast cells were then serially diluted (cells/ml) and transferred to a medium without histidine, leucine, tryptophan, and adenine to assess protein interaction. For the co‐IP assay, ATG8‐mCherry or free mCherry was co‐expressed with DCL2‐eGFP or AGL1‐eGFP in *F. graminearum*. After protein expression was confirmed by western blotting, total protein was extracted from fungal strains. Co‐IP was carried out using GFP beads, as described previously.^[^ ^115^ ^]^ Eluted proteins were analyzed by western blotting.

### LC‐MS‐MS Analysis

Samples were digested on beads with trypsin at 37 °C overnight and then desalted using StageTip C18 tips (Thermo Fisher Scientific, USA). The resulting peptides were separated on an Ultimate 3000 nano UHPLC system (Thermo Fisher Scientific, USA) and analyzed by a hybrid Quadrupole‐Orbitrap mass spectrometer (Q Exactive HF, Thermo Fisher Scientific) in the data‐dependent acquisition mode.^[^ ^116^ ^]^ The raw data were processed using the Sequest search mode in Proteome Discoverer version 3.1.1.93 (Thermo Fisher Scientific).

### Western Blot Assay

Western blotting was carried out as described previously.^[^ ^115^ ^]^ For the detection of MBP‐, GST‐, GFP‐ and mCherry‐tagged proteins, anti‐MBP (1:5000; TransGen Biotech), anti‐GST (1:5000; TransGen Biotech), anti‐GFP (1:5000; polyclonal antibody prepared in‐house), anti‐mCherry (1:5000; Proteintech, China), and secondary goat anti‐mouse immunoglobulin G‐horseradish peroxidase (IgG‐HRP) (1:5000; Proteintech) antibodies were used.

### FMN Reductase Activity Assays

Enzymatic activity was assayed as previously described^[^ ^68^ ^]^ with slight modifications. GST‐FMR1, GST‐YLR011wp, GST, MBP‐p29, and MBP were expressed in *E. coli* and purified. GST‐YLR011wp (0.25 µM) or GST‐FMR1 (0.25 µM) plus MBP‐p29 (0.25 µM) or MBP (0.25 µM) was incubated with NAD(P)H (150 µM) in 50 mM Tris‐HCl (pH 7.6) and FMN (100 µM). The oxidation of NAD(P)H was monitored by measuring the decrease in absorption at 340 nm using Varioskan LUX Multifunctional enzyme marker (Thermo Fisher Scientific). Commercial FMN reductase (MedChemexpress, USA) was included in the experiment as a control.

### Pathogenicity Assays, ROS Detection and ROS Inhibitor Treatment

The pathogenicity of *F. graminearum* strains in wheat leaves and heads was assessed as described previously.^[^ ^117^ ^]^ DAB and NBT staining and chemiluminescent assay were performed as described previously.^[^ ^117^, ^118^ ^]^ For ROS inhibition experiment, NAC (MedChemExpress, USA) was added to PDA medium (5 mM) as described previously.^[^ ^119^ ^]^

### Autophagy Experiments

MDC **s**taining was carried out as described previously.^[^ ^120^ ^]^ 3‐MA, rapamycin, and MG132 treatments followed previously described methods.^[^ ^121^ ^]^

### Fluorescence Signal Imaging

The subcellular localization of fluorescence signals was observed with a confocal laser scanning microscope (Olympus FV3000, Japan or Zeiss LSM 880, Germany). GFP was excited at 488 nm and captured at 510–550 nm. mCherry was excited at 561 nm and captured at 561–630 nm. The MDC staining signal was excited at 405 nm and captured at 635–708 nm.

### Statistical Analysis

Data were presented as mean ± SD (*n* = 3 or 5 for each analysis). Statistical comparisons were performed using Student's *t*‐test for comparisons between two groups or ANOVA (Analysis of Variance) followed by Tukey's Honestly Significant Difference (HSD) post‐hoc test for comparisons among three or more groups. A p‐value (*P*) < 0.05 was considered statistically significant. All analyses were conducted using IBM SPSS Statistics version 20 (New York, USA).

## Conflict of Interest

The authors declare no conflict of interest.

## Author Contributions

L.S. and S.Z. designed research; S.Z., T.P., S.P., S.Z., and Z.D. performed research; S.Z., T.P., N.S., Z.K., and I.B.A. analyzed data; I.B.A., and L.S. wrote the manuscript.

## Supporting information



Supporting Information

## Data Availability

The authors declare that the data supporting the findings of this study are available within the paper, its supplementary information files.
