# Supplementary material for: A Viral RNA Silencing Suppressor Modulates Reactive Oxygen Species Levels to Induce the Autophagic Degradation of Dicer‐Like and Argonaute‐Like Proteins
Source: Adv Sci (Weinh). 2025 Sep 8;12(44):e06572. doi: 10.1002/advs.202506572 (PMC12667476; doi:10.1002/advs.202506572)
Supplement: Supplementary file 1 — Supporting Information [file ADVS-12-e06572-s001.pdf]

Supplemental Information (SI) Appendix

A Viral RNA Silencing Suppressor Modulates Reactive Oxygen Species Levels to Induce the Autophagic Degradation of Dicer-like and Argonaute-like Proteins

Shiyu Zhai[1], Tianxing Pang[1], Shiyu Peng[1], Shenshen Zou[3], Zhiping Deng[4], Nobuhiro Suzuki[5], Zhensheng Kang[1], Ida Bagus Andika[1 *], Liying Sun[1,2 *]

[1] State Key Laboratory of Crop Stress Biology for Arid Areas and College of Plant Protection, Northwest A&F University, Yangling, 712100, Shaanxi, China

[2] Institute of Future Agriculture, Northwest A&F University, Yangling 712100, Shaanxi, China

[3] Department of Plant Pathology, College of Plant Protection, Shandong Agricultural University, Tai'an, China

[4] Institute of Virology and Biotechnology, Zhejiang Academy of Agricultural Sciences, Hangzhou, Zhejiang 310021, PR China

[5] Institute of Plant Science and Resources (IPSR), Okayama University, Kurashiki, 710-0046, Japan

Includes:

SI Figures, Figure S1-S9

SI Tables, Table S1-S4

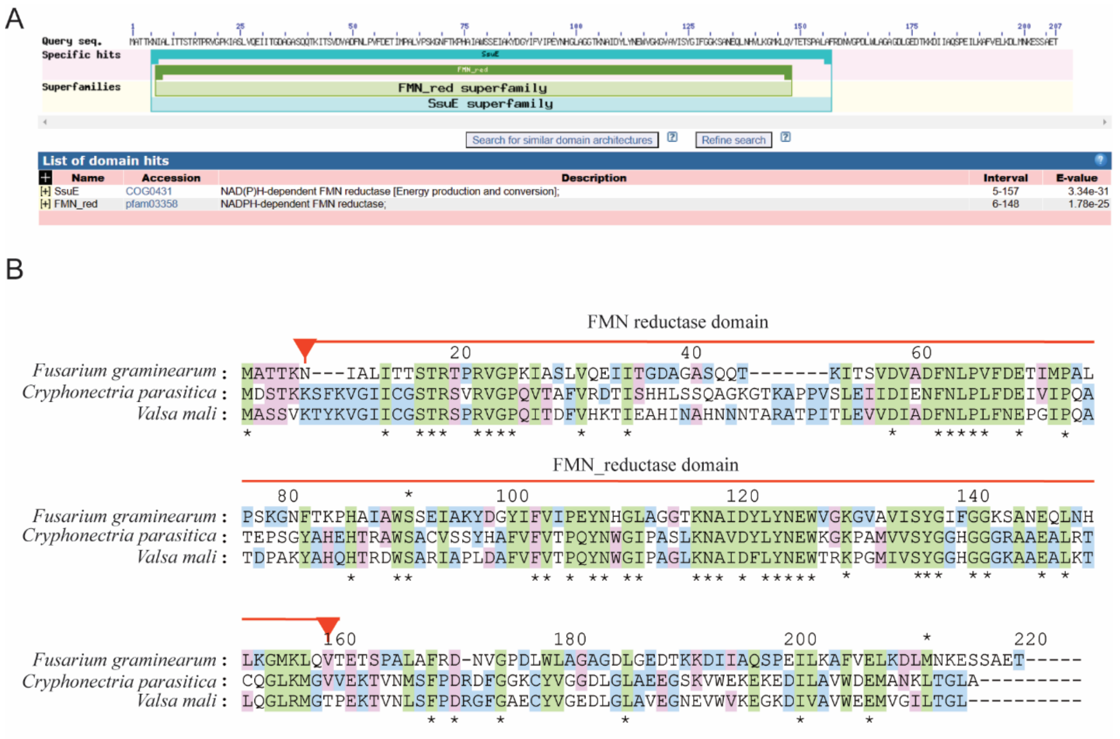

Figure S1. Sequence characterization of FMR1. **A)** Results of a conserved domain database search (NCBI) using the FMR1 sequence. **B)** Amino acid sequence alignment of FMR1 homologs encoded by *F. graminearum*, *C. parasitica*, and *V. mali*.

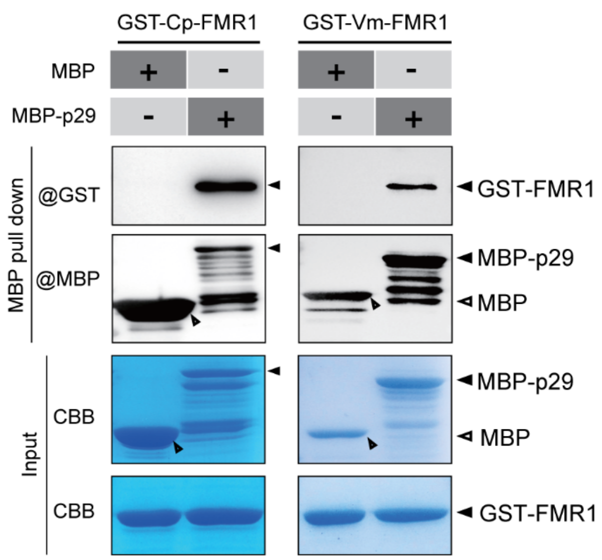

Figure S2. Interaction of p29 with *C. parasitica* and *V. mali* FMR1 in an *in vitro* pull-down assay. Prokaryotically expressed MBP-p29 was incubated with GST-FMR1, after which MBP pull-down was performed. Protein samples after the MBP pull-downs were subjected to immunoblotting with anti-MBP and anti-GST antibodies. Expressed fusion proteins were run on sodium dodecyl sulfate-polyacrylamide gel electrophoresis (SDS-PAGE) and stained with Coomassie brilliant blue (CBB).

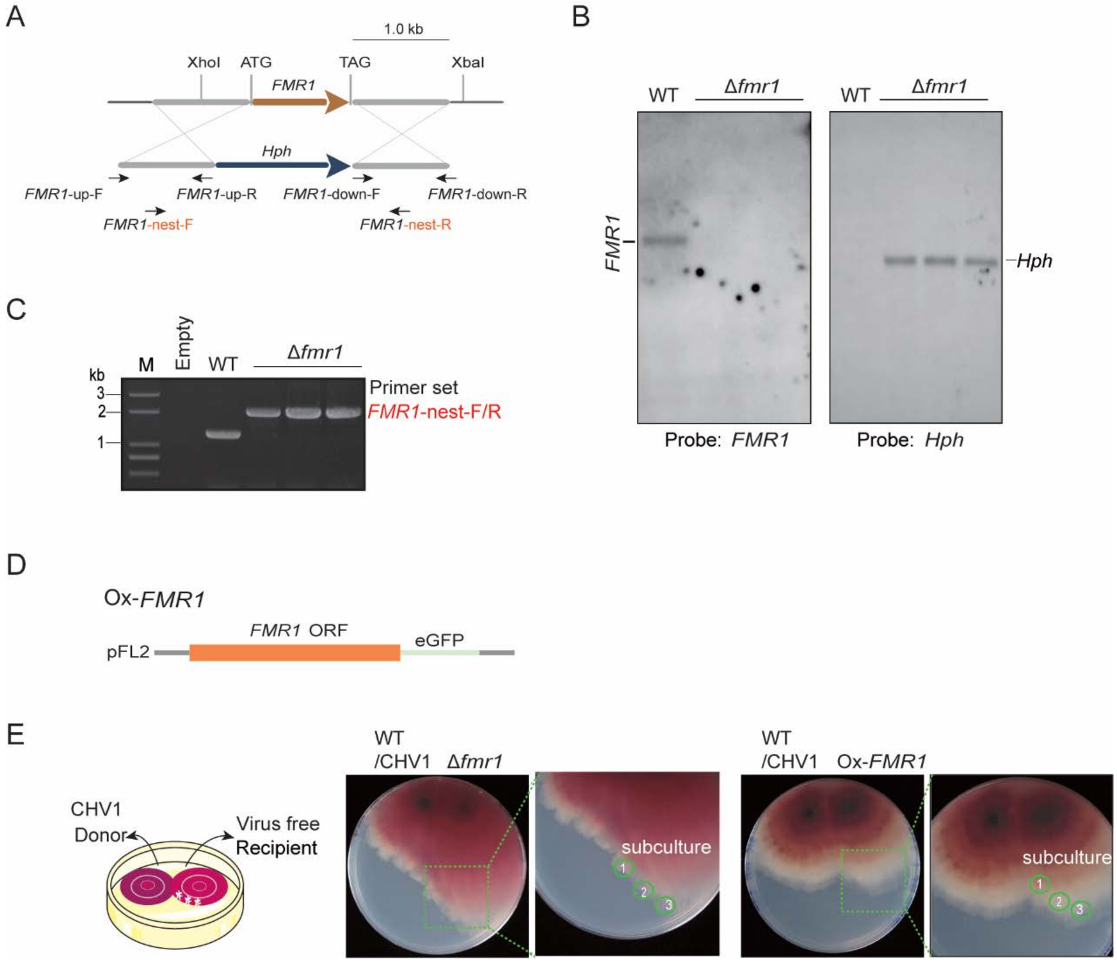

Figure S3. Generation of *F. graminearum FMR1*-disrupted mutant (Δ*fmr1*) and *FMR1*-overexpressing (Ox-*FMR1*) strains. **A)** Schematic diagram of the DNA segment used to disrupt the *FMR1* gene through homologous recombination. **B)** Southern blot analysis of the Δ*fmr1* strain. **C)** Genomic PCR analysis of the Δ*fmr1* strain. **D)** Schematic diagram of the plasmid construct used to generate Ox-*FMR1*. **E)** Introduction of CHV1 to Δ*fmr1* and Ox-*FMR1* strains through hyphal anastomosis.

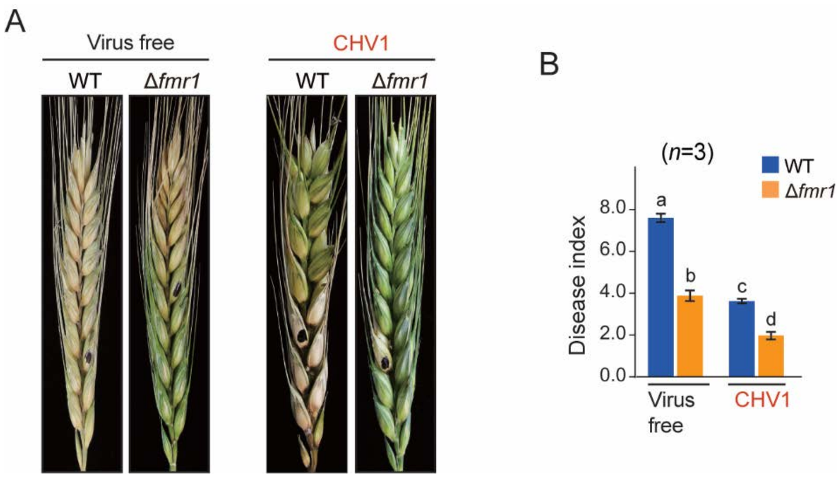

Figure S4. Pathogenicity assay of WT and Δ*fmr1* strains. **A)** Inoculation of fungal conidia on wheat heads. Photographs were taken 7 days after inoculation. **B)** Calculated disease index based on observations from the experiment described in A. Error bars represent the mean ± standard deviation (SD) of three biological replicates. Different letters indicate significant differences ($P < 0.05$, one-way ANOVA, Tukey’s HSD test).

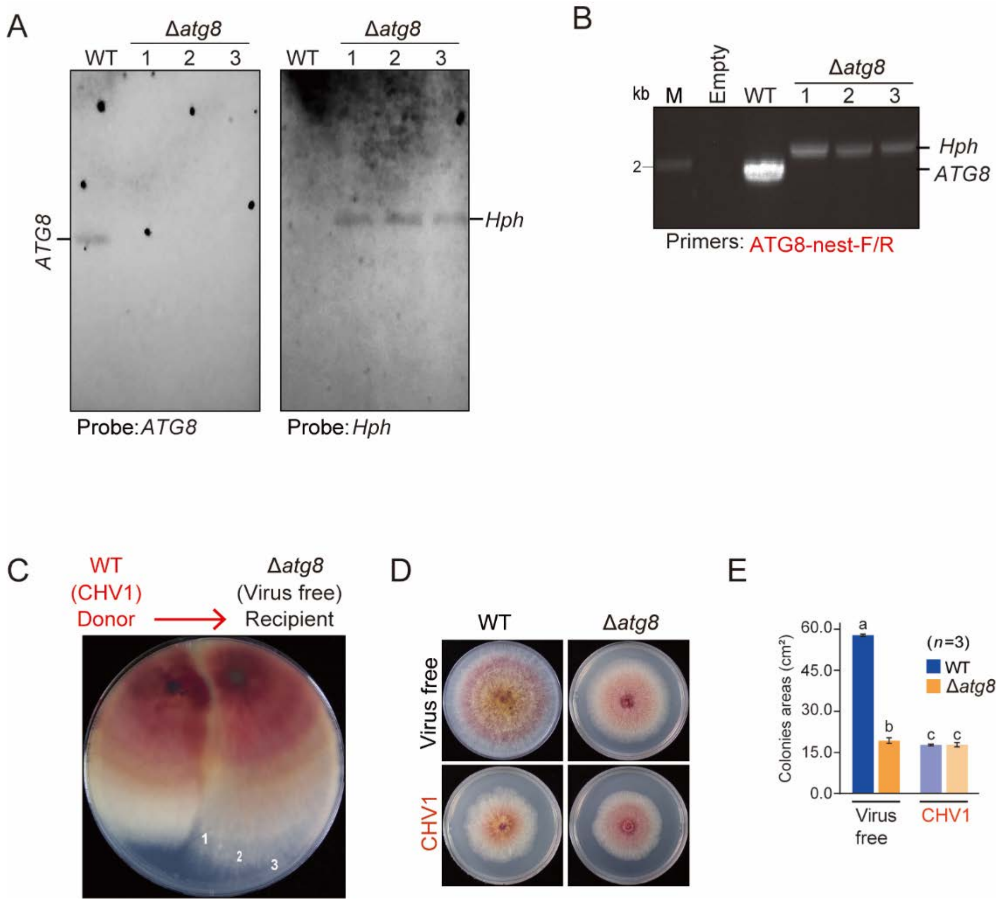

Figure S5. Generation of the *F. graminearum ATG8* knockout strain (Δ*atg8*). **A)** Southern blot analysis of the Δ*atg8* strain. **B)** Genomic PCR analysis of the Δ*atg8* strain. **C)** Introduction of CHV1 to the Δ*atg8* strain through hyphal anastomosis. **D)** Colony growth and morphology of the Δ*atg8* strain with or without CHV1 infection. **E)** Fungal colony sizes observed in the experiment described in D. Error bars indicate the mean ± standard deviation (SD) of three biological replicates. Different letters indicate significant differences ($P < 0.05$, one-way ANOVA, Tukey's HSD test).

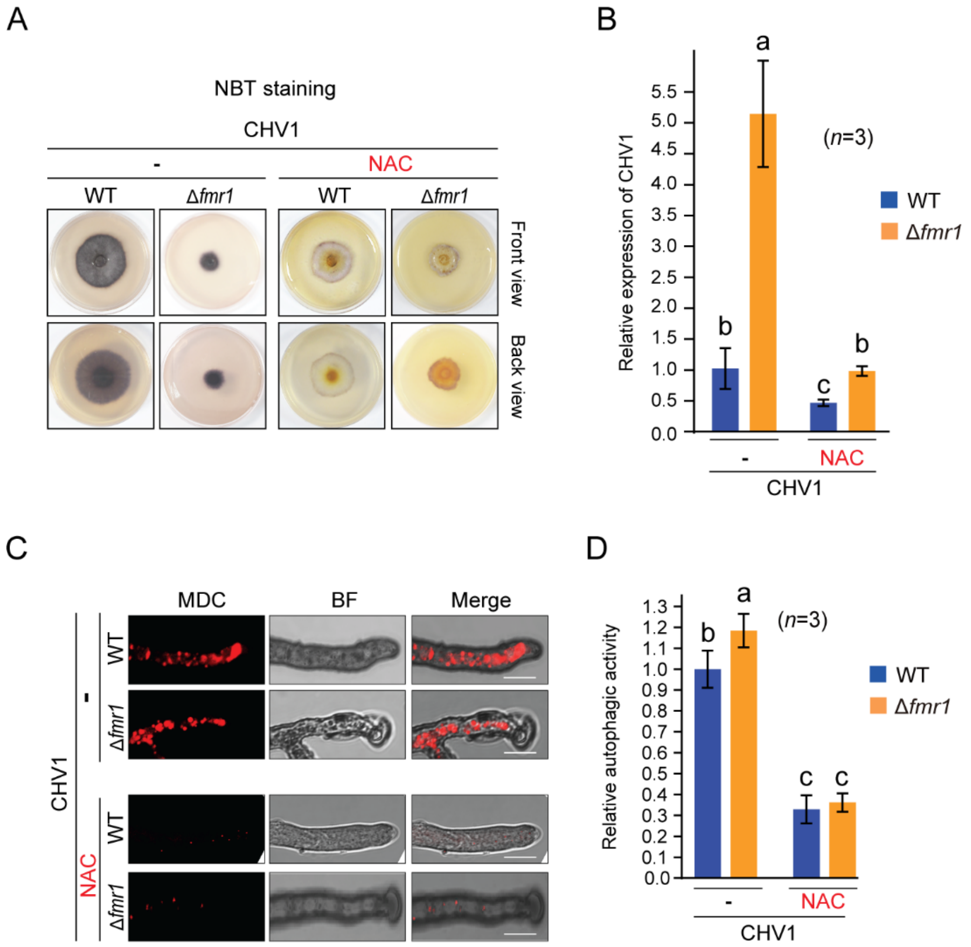

Figure S6. Treatment of *F. graminearum* wild-type (WT) and Δ*fmr1* strains with the ROS inhibitor N-acetyl-L-cysteine (NAC). **A)** Staining of fungal mycelia grown on PDA using NBT to visualize $O_2^{\bullet -}$ accumulation. Staining was done 3 days after culturing. The experiments were repeated three times, with three replicates each time. **B)** CHV1 RNA accumulation levels in WT and Δ*fmr1* strains analyzed by qRT-PCR. The WT untreated sample value was set to 1.0. Error bars represent the mean ± standard deviation (SD) of three biological replicates. Different letters indicate significant differences ($P < 0.05$, one-way ANOVA, Tukey's HSD test). **C)** Staining of autophagosomes in the mycelia of WT and Δ*fmr1* strains using the fluorescent dye MDC. Fluorescence signals in mycelial cells were observed by confocal laser scanning microscopy. Scale bar, 10 μm. **D)** Relative autophagic activity based on the number of MDC-stained bodies observed in the experiment described in C. Each bar represents the mean number of structures counted from 100 cells obtained from three independent experiments. The WT untreated sample value was set to 1.0. Error bars represent the mean ± standard deviation (SD) of three biological replicates. Different letters indicate significant differences ($P < 0.05$, one-way ANOVA, Tukey's HSD test).

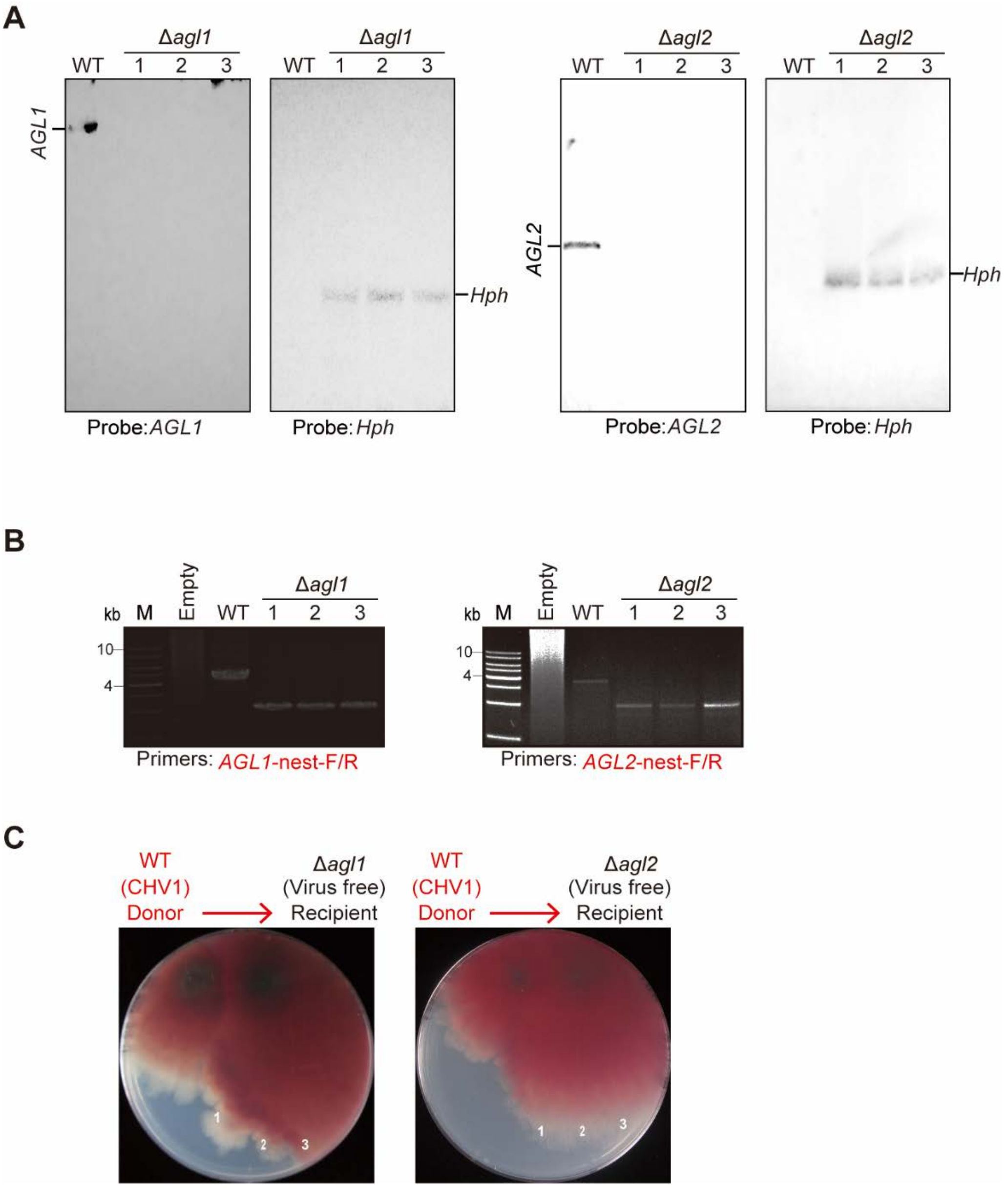

Figure S7. Generation of *F. graminearum AGL1 and AGL2* knockout strains (Δ*agl1* and Δ*agl2*). **A)** Southern blot analysis of Δ*agl1* and Δ*agl2* strains. **B)** Genomic PCR analysis of Δ*agl1* and Δ*agl2* strains. **C)** Introduction of CHV1 to Δ*agl1* and Δ*agl2* strains through hyphal anastomosis.

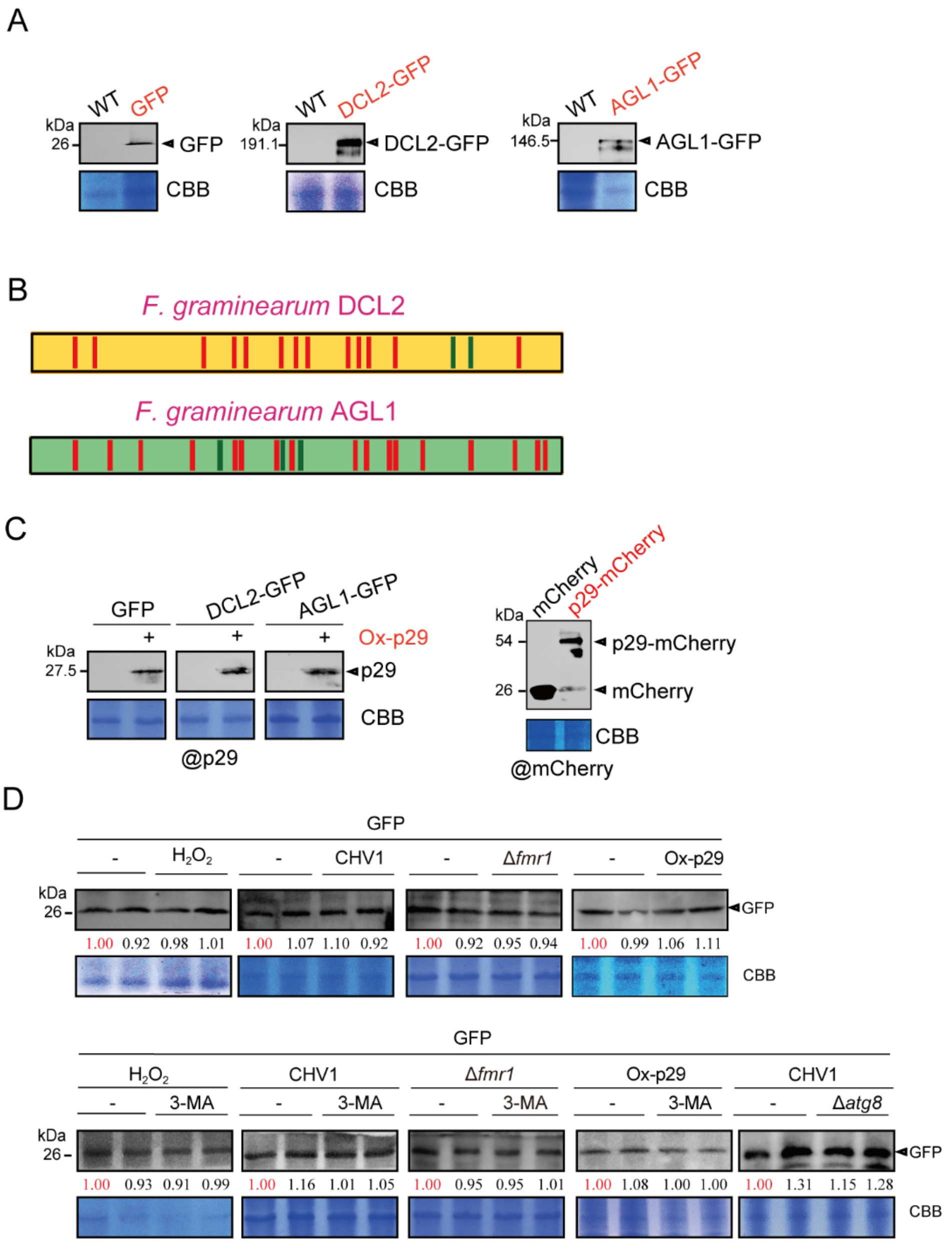

Figure S8. Western blot analysis of the accumulation of proteins expressed in *F. graminearum* strains. **A)** Western blot detection of GFP, DCL2-GFP, and AGL1-GFP accumulation using an anti-GFP antibody. **B)** A schematic diagram showing a potential ATG8-interacting motif (AIM) in DCL2 and AGL1 (not to scale). Red lines indicate the relative position of the AIM in the proteins. The AIM was predicted using an online bioinformatics analysis tool (http://repeat.biol.ucy.ac.cy/iLIR). **C)** Western blot detection of p29 and p29-mCherry accumulations using an anti-p29 and -mCherry antibodies. **D)** Western blot detection of GFP accumulation using an anti-GFP antibody.

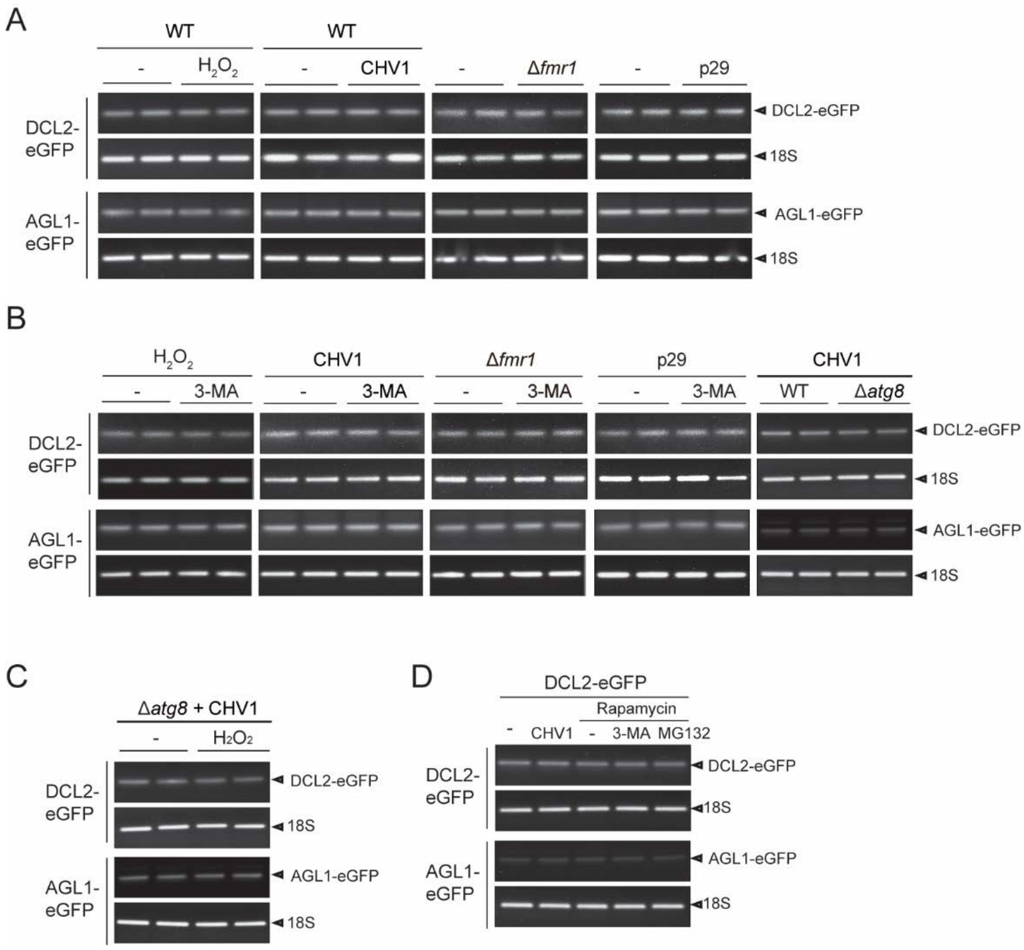

Figure S9. RT-PCR detection of DCL2-GFP and AGL1-GFP transcripts in the fungal strains used for the experiments described in Fig. 6A–D.

Table S1. The p29-interacting protein candidates identified.

Accession No.	Protein name	Predicted function/domain
XP_011322075.1	hypothetical protein FGSG_12402	NAD(P)H-dependent FMN reductase
XP_011321751.1	hypothetical protein FGSG_03917	Pyrroline-5-carboxylate reductase
XP_011325209.1	hypothetical protein FGSG_11142	Predicted dehydrogenase
XP_011328564.1	hypothetical protein FGSG_09211	FtsJ-like methyltransferase
XP_011316640.1	hypothetical protein FGSG_00905	Putative S-adenosyl-L-methionine-dependent methyltransferase
XP_011327224.1	AdoMet-dependent rRNA methyltransferase spb-1	23S rRNA U2552 (ribose-2'-O)-methylase RlmE/FtsJ
XP_011318196.1	hypothetical protein FGSG_02288	CCCH-type zinc finger
XP_011318008.1	hypothetical protein FGSG_02130	NF-X1-zinc-finger
XP_011318988.1	hypothetical protein FGSG_10059	DEXH-box helicase domain of DEAD box protein 60
XP_011321198.1	hypothetical protein FGSG_04408	DEAD-like helicase

Table S2. Primers used in this study.

Clone Name	Primer Name	Oligonucleotide sequence (5’- 3’)
**Primers used for bacterial expression**		
pMAL-MBP-p29	F_{XbaI}-MBPp29	tcagaattcggatcctctagaATGGCTCAATTAAGAAAACC
	R_{HindIII}-MBPp29	acgacggccagtgccaagcttCTAGCCAATCCGGGCAAGGGGA
pGEX-4T-1-FgFMR1	F_{EcoRI}-GSTFgFMR1	tggatccccggaattcATGGCGACCACGAAGAATAT
	R_{EcoRI}-GSTFgFMR1	gtcgacccgggaattcCTAAGTCTCTGCTGATGATT
pGEX-4T-1-CpFMR1	F_{EcoRI}-GSTCpFMR1	tggatccccggaattcATGGATTCTACCAAGAAATC
	R_{EcoRI}-GSTCpFMR1	gtcgacccgggaattcTCAAGCAAGTCCCGTCAGCTTGT
pGEX-4T-1-VmFMR1	F_{EcoRI}-GSTVmFMR1	tggatccccggaattcATGGCATCCTCAGTAAAGAC
	R_{EcoRI}-GSTVmFMR1	gtcgacccgggaattcTCACCCGGTCAGAATCCCAACCA
pGEX-4T-1-FgFMR1(D)	F_{EcoRI}-GSTFgFMR1(D)	tggatccccggaattcAT**G**AATATCGCGCTTATCACAACGA
	R_{EcoRI}-GSTFgFMR1(D)	gtcgacccgggaattcCTACACCTGAAGTTTCATACCCTTT
pGEX-4T-1-YLR011wp	F_{EcoRI}-YLR011wp	tggatccccggaattcATGAAAGTGGGTATTATAATGGG
	R_{EcoRI}-YLR011wp	gtcgacccgggaattcTTATTTATTCCTCGTTGTTTCGATG
**Primers used for yeast two-hybrid**		
pGBKT7-p29	F_{EcoRI}-p29	atggccatggaggccgaattcATGGCTCAATTAAGAAAACC
	R_{BamHI}-p29	ccgctgcaggtcgacggatccCTAGCCAATCCGGGCAAGGGGA
pGADT7-FgFMR1	F_{EcoRI}-FgFMR1	gccatggaggccagtgaattcATGGCGACCACGAAGAATAT
	R_{BamHI}-FgFMR1	cagctcgagctcgatggatccCTAAGTCTCTGCTGATGATT
pGADT7-CpFMR1	F_{EcoRI}-CpFMR1	gccatggaggccagtgaattcATGGATTCTACCAAGAAATC
	R_{BamHI}-CpFMR1	cagctcgagctcgatggatccTCAAGCAAGTCCCGTCAGCTTGT
pGADT7-VmFMR1	F_{EcoRI}-VmFMR1	gccatggaggccagtgaattcATGGCATCCTCAGTAAAGAC
	R_{BamHI}-VmFMR1	cagctcgagctcgatggatccTCACCCGGTCAGAATCCCAACCA
**Primers used for over expression**		
pDL2/pFL2-FgDCL2	F_{XhoI}-FgDCL2	tcaccatcaccatcactcgagATGTCCTCAAGCGATAAGGTCATGGCG
	R_{XhoI}-FgDCL2	cagctcctcgcccttgctcacAATGAGTTCCATGGCAACAAGAAATCG
pDL2/pFL2-FgAGL1	F_{XhoI}-FgAGL1	tcaccatcaccatcactcgagATGGCGGACAGAGGTGATCGAGGCGG
	R_{XhoI}-FgAGL1	cagctcctcgcccttgctcacGATATAGTACATCGAGTTGGCGAGGT
pDL2/pFL2-p29-mCherry	F_{XhoI}-p29-mCherry	tcaccatcaccatcactcgagATGGCTCAATTAAGAAAACC
	R-p29-mCherry	tcctcctcgcccttgctcaccatGCCAATCCGGGCAAGGGGATC
	F-p29-mCherry	GATCCCCTTGCCCGGATTGGCatggtgagcaagggcgaggagga
	R_{XhoI}-p29-mCherry/	cagctcctcgcccttgctcacTCACTTGTACAGCTCGTCCAT
pDL2/pFL2-p29	F_{XhoI}-p29	tcaccatcaccatcactcgagATGGCTCAATTAAGAAAACC
	R_{XhoI}-p29/	cagctcctcgcccttgctcacTTAGCCAATCCGGGCAAGGGGA
pDL2/pFL2-FgATG8-mCherry	F_{XhoI}-FgATG8-mCherry	tcaccatcaccatcactcgagATGCGCAGCAAATTCAAGGAC

	R-FgATG8-mCherry	cccttgctcaccatCGCTTCGCCAAAAG
	F-FgATG8-mCherry	CTTTTGGCGAAGCGatggtgagcaaggg
	R*XhoI*-FgATG8-mCherry/	cagctcctcgcccttgctcacTCACTTGTACAGCTCGTCCAT
pDL2/pFL2-FgFMR1	F*XhoI*-FgFMR1	tcaccatcaccatcactcgagATGGCGACCACGAAGAATAT
	R*XhoI*-FgFMR1	cagctcctcgcccttgctcacAGTCTCTGCTGATGATT
pDL2/pFL2-mCherry	F*XhoI*-mCherry	tcaccatcaccatcactcgagATGGTGAGCAAGGGCGAGGA
	R*XhoI*-mCherry/	cagctcctcgcccttgctcacTCACTTGTACAGCTCGTCCAT
GFP-ATG8	F-pHD64-XhoI-ProAtg8	TGGTTCTCATCACCATCACCATCACTCGAGACTGAGAACTCGGG TGATAGTCAGA
	R-ProAtg8-GFP	gtgaacagctcctcgcccttgctcaccatCATGTTGACGGTGATGGTTGTTGTGG A
	F-ProAtg8-GFP	ACACTTACCGCCTCCACAACAACCATCACCGTCAACatggtgagcaa gggcgaggag
	R-GFP-FgAtg8	cgaaggggtgctcgtccttgaatttgctgcgCTTGTACAGCTCGTCCATGCCGAG AGT
	F-GFP-FgAtg8	GGGATCACTCTCGGCATGGACGAGCTGTACAAGatgcgcagcaaattca aggacg
	R-pHD64-FgAtg8-XhoI	TTGAGTGGAATGATGGGATCCAAGCTCGAGTTACGCTTCGCCA AAAGTGTTCTCG
mCherry-ATG8	F-pHD64-XhoI-ProAtg8	TGGTTCTCATCACCATCACCATCACTCGAGACTGAGAACTCGGG TGATAGTCAGA
	R-ProAtg8-mCherry	ccatgttatcctcctcgcccttgctcaaCATGTTGACGGTGATGGTTGTTGTGGA
	F-ProAtg8-mCherry	ACACTTACCGCCTCCACAACAACCATCACCGTCAACatgttgagcaag ggcgagg
	R-mCherry-FgAtg8	cgaaggggtgctcgtccttgaatttgctgcgCATCTTGTACAGCTCGTCCATGCC
	F-mCherry-FgAtg8	CACTCCACCGGCGGCATGGACGAGCTGTACAAGatgcgcagcaaattca aggacg
	R-pHD64-FgAtg8-XhoI	TTGAGTGGAATGATGGGATCCAAGCTCGAGTTACGCTTCGCCA AAAGTGTTCTCG
**Primers used for knock out**		
Hph	F-Hph	ggaggtcaacacatcaatgcctatt
	R-Hph	ctactctattcctttgccctcggac
knock out Fg*fmr1*	F-Up-Fg*fmr1*	ATCGCCTCTCTTTGGAAAGGGCTTC
	R-Up-Fg*fmr1*	caaaataggcattgatgtgttgacctccTGTATCTAAACTGTTTGAAGA
	F-DOWN-Fg*fmr1*	ctcgtccgagggcaaaggaatagagtagAGTAGATGACCAGACAGTATA
	R-DOWN-Fg*fmr1*	TAGTGTGACACCCTTTTGCAT
	F- Fg*fmr1*-nest	ATCAAGTCAGGACATCCCGAACCGCG
	R- Fg*fmr1*-nest	TGAGCCCACATACCAACTGACTGCGC
knock out Fg*atg8*	F-Up-Fg*atg8*	CTCCTTTATATGAGAAGCAGATGCCATC

	R-Up-Fg*atg8*	caaaataggcattgatgtgttgacctccGGTTACAGTGGGCGCCCAGGGCAGA
	F-DOWN-Fg*atg8*	ctcgtccgagggcaaaggaatagagtagTATGACAATGTCTGGCCCTT
	R-DOWN-Fg*atg8*	TCCCAAACAAAGCGCTGTTCTTCGAAAA
	F-Fg*atg8*-nest	CTTGATGGAGCCCTTTCCAATCC
	R-Fg*atg8*-nest	TATTCTCGATGACTCTTCGTTCA
knock out Fg*agl1*	F-Up-Fg	ATGTTGTGATGCTATCACAAGTGTGC
	R-Up-Fg	caaaataggcattgatgtgttgacctccGATGAGATAATGTCTGGGTA
	F-DOWN-Fg	ctcgtccgagggcaaaggaatagagtagGTGTGCCGTGTGTGGGAGAT
	R-DOWN-Fg	CGCCGAAGCAGCCGCCTCTTTTGGTCGC
	F-Fg*agl1*-nest	AGCGCTGCTGTTGCTGATTTGCTTTGGC
	R-Fg*agl1*-nest	ATCGCATCTGCTTCTTATACGATTAATC
knock out Fg*agl2*	F-Up-Fg*agl2*	ATGTCACAGTGCTTTGATATAAACAATAA
	R-Up-Fg*agl2*	caaaataggcattgatgtgttgacctccGGTGACAGATGAGTCTGAAG
	F-DOWN-Fg*agl2*	ctcgtccgagggcaaaggaatagagtagCCAGAACAATGGCGTTGATG
	R-DOWN-Fg*agl2*	TCTCATCGAGGTCATTTCATCGACGTTAG
	F-Fg*agl2*-nest	AGCCCCTGTTGCCCTACATCACATT
	R-Fg*agl2*-nest	TCACATGGTTAGAATTGATCTCGAACT
**Primers used for RT-qPCR**		
	F-p29-RT	TTGTAGACAGCGCAAGATGG
	R-p29-RT	CCCAAAATGGCTTGGTAGAA
	F-CHVI-RT	TGAGAGATTGGCAGGGGGAAACCC
	R-CHV1-RT	TGTCTGAATCCTACTGCAGCGGCC
	F-Fg18s-RT	AGGCAATAACAGGTCTGTGATGCC
	R-Fg18s-RT	TGAGCCATTCAATCGGTAGTAGCG
	F-Fg*fmr1*-RT	ATTAGCTATGGTATCTTTGGAGGGAAAT
	R-Fg*fmr1*-RT	CTAAGTCTCTGCTGATGATTCCTTGTTC
	F-Cp18s-RT	ATAACAGGTCTGTGATGCCCTT
	R-Cp18s-RT	CTCGCTGGCTCTGTCAGTGTAG
	F-Cp*fmr1*-RT	GTTCAAGGTCGGCATCATTT
	R-Cp*fmr1*-RT	CGAAGAGGGGGAGGTTAAAG
	F-Vm18s-RT	AAACTTTCAACAACGGATCTCTTGG
	R-Vm18s-RT	AAATGACGCTCGAACAGGCATG
	F-Vm*fmr1*-RT	GGCATGATTGTGTCGTATGG
	R-Vm*fmr1*-RT	TCAGAATCCCAACCATCTCC
	F-DCL2GFP-RT	GCGTGAATTTTTTGAATGGGGAGC
	R-DCL2GFP-RT	GGACACGCTGAACTTGTGGCCGTT
	F-AGL1GFP-RT	GTTGAAAGTGTCTCAACAGCTGGG

	R-AGL1GFP-RT	CACGCTGAACTTGTGGCCGTTTAC
	F-Fg*dcl1*-RT	TTCCAAGTCTCGACTAACGG
	R-Fg*dcl1*-RT	AGGTGACACGTTGAGGGGTT
	F-Fg*dcl2*-RT	GCGAACCAGTGCCTACAGAA
	R-Fg*dcl2*-RT	AAGACGAGCGAGAAGTGTCC
	F-Fg*agl1*-RT	AAGATATCCGGTAACTGTGCCAAGC
	R-Fg*agl1*-RT	ACGACAGGCTCGTTCAATCTCAGGT
	F-Fg*agl2*-RT	AGATCGGTCAAGATCCTTATCTGAG
	R-Fg*agl2*-RT	TGGTCTTGAATGTTGTGGCGAATGCT
**Primers used for Southern blot**		
	F-Hph	GGAGGTCAACACATCAATGCCTATT
	R-Hph	CTACTCTATTCCTTTGCCCT
	F-Fg*fmr1*	GCGACCACGAAGAATATCGCGCTTATCA
	R-Fg*fmr1*	TGATAAGCGCGATATTCTTCGTGGTCGC
	F-Fg*atg8*	ATGCGCAGCAAATTCAAGGACGAGC
	R-Fg*atg8*	TTACGCTTCGCCAAAAGTGTTCTCG
	F-Fg*agl1*	ATGGGAGAGGACGAGGTGATGGTGG
	R-Fg*agl1*	AACTCGTTGTGGAGCTTGTGGTTTA
	F-Fg*agl2*	ATGTCTGATAGAGGGCGCTCACCCTCTCCA
	R-Fg*agl2*	TTAGATGTACCACATGGTACTGCGGAAAAA

Table S3. Plasmid constructs generated in this study.

Construct [a]	Gene[b]	Plasmid[c]	Restriction sites
MBP-p29	p29	pMAL-c2X-MBP	*Xba*I and *Hind*III
GST-FgFMR1	FgFMR1	pGEX-4T-1	*EcoR*I
GST-CpFMR1	CpFMR1	pGEX-4T-1	*EcoR*I
GST-VmFMR1	VmFMR1	pGEX-4T-1	*EcoR*I
GST-YLR011wp	YLR011wp	pGEX-4T-1	*EcoR*I
GST-FgFMR1(D)	FgFMR1(D)	pGEX-4T-1	*EcoR*I
FgFMR1-GFP	FgFMR1	pFL2	*Xho*I
p29-mCherry	p29-mCherry	pDL2	*Xho*I
FgDCL2-GFP	FgDCL2	pDL2/pFL2	*Xho*I
FgAGL1-GFP	FgAGL1	pDL2/pFL2	*Xho*I
GFP-FgATG8	GFP-FgATG8	pHD64	*Xho*I
mCherry-FgATG8	mCherry-FgATG8	pHD64	*Xho*I
FgATG8-mCherry	FgATG8-mCherry	pFL2	*Xho*I
Oxp29	p29	pDL2/pFL2	*Xho*I
BD-p29	p29	pGBKT7	*EcoR*I *and BamH*I
AD-FgFMR1	FgFMR1	pGADT7	*EcoR*I *and BamH*I
AD-CpFMR1	CpFMR1	pGADT7	*EcoR*I *and BamH*I
AD-VmFMR1	VmFMR1	pGADT7	*EcoR*I *and BamH*I

[a]Construction of plasmid vectors:

All plasmid vectors were constructed using homologous recombination with the One Step Cloning Kit (Vazyme Biotech, China).

1. Prokaryotic expression vectors

The coding regions of p29, FgFMR1, CpFMR1, VmFMR1, YLR011wp, and FgFMR1(D) were

amplified by RT-PCR and inserted into either:

- The pMAL-c2X-MBP plasmid (digested with *XbaI* and *HindIII*), or
- The pGEX-4T-1 plasmid (digested with *EcoRI*).

2. Fungal expression vectors

- The coding regions of FgFMR1, FgDCL2, and FgAGL1 were amplified by RT-PCR and inserted into GFP-carrying pDL2 or pFL2 plasmids (digested with *Xho*I).
- The p29-mCherry and FgATG8-mCherry fusions were generated by overlapping PCR and inserted into pDL2 or pFL2. A stop codon was added to disable GFP expression.
- FgATG8promoter:GFP-FgATG8 and FgATG8promoter:mCherry-FgATG8 fusions were generated by overlapping PCR and inserted into the pHD64 plasmid (digested with *Xho*I).
- The p29 coding region was amplified by PCR and inserted into pDL2 or pFL2, with a stop codon added to terminate translation.

3. Yeast two-hybrid vectors

The coding regions of p29, FgFMR1, CpFMR1, and VmFMR1 were amplified by RT-PCR and inserted into either pGBKT7 or pGADT7 (digested with *EcoR*I and *BamH*I).

[b]**Accession number**:

FgFMR1(XP_011322075.1), CpFMR1(XM_040923474.1), VmFMR1(KUI70844.1), FgDCL2 (XP_011321198.1), FgAGL1(XM_009260855.1), FgATG8 (XM_011327366.1), YLR011wp(NP_013111.1).

[c]**Plasmid references**:

pDL2/ pFL2 [1] ; pHD64 [2].

1. Zhou X, Li G, Xu J-R. Efficient approaches for generating gfp fusion and epitope-tagging constructs in filamentous fungi. *Fungal genomics: Methods and protocols*. 2011:199-212
2. Sun F, Zhang R, Li T *et al.* Fusarium graminearum gga protein is critical for fungal development, virulence and ascospore discharge through its involvement in vesicular trafficking. *Environmental Microbiology*. 2022;**24**:6290-306

Table S4. Abbreviations in this study.

Abbreviations	Full name
AGO	Argonaute
AGL	Argonaute - like
ATG	Autophagy - related
CBB	Coomassie brilliant blue
CHV1	Cryphonectria hypovirus 1
CMAC	7-amino-4-chloromethylcoumarin
CO - IP	Co-immunoprecipitation
C. parasitica	*Cryphonectria parasitica*
DAB	3,3'-Diaminobenzidine
DCL	Dicer - like
F. graminearum	*Fusarium graminearum*
FMN	Flavin mononucleotide
FMR1	NAD(P)H - dependent FMN reductase 1
GFP	Green fluorescent protein
GST	Glutathione S - transferase
H_2O_2	Hydrogen peroxide
lgG -HRP	Immunoglobulin G - horseradish peroxidase
LC - MS - MS	Liquid chromatography with tandem mass spectrometry
MBP	Maltose-binding protein
mCherry	Monomeric cherry red fluorescent protein
MDC	Monodansylcadaverine
MG-132	Carbobenzoxy-leucyl-leucyl-leucinal
NAC	N-acetyl-L-cysteine
NBT	Nitroblue tetrazolium
$OH^{\cdot}$	Hydroxyl radical
ORFs	Open reading frames
Ox	Overexpressing
$O_2^{\cdot -}$	Superoxide radical
O_2	Singlet oxygen
PDA	Potato dextrose agar
qRT - PCR	Quantitative real-time PCR
RNAi	RNA interference
ROS	Reactive oxygen species
RT - PCR	Reverse transcription-polymerase chain reaction
SDS - PAGE	Sodium dodecyl sulfate - polyacrylamide gel electrophoresis
V. mali	*Valsa mal*i
WT	Wild - type
3 - MA	3-Methyladenine
